# Synthesis of novel phenoxyacetohydrazide compounds and evaluation therapeutic potential exhibit as anti-inflammatory and anti-angiogenic

**DOI:** 10.1371/journal.pone.0330731

**Published:** 2025-09-26

**Authors:** Yasser Hussein Issa Mohammed, Ahmed Hassen Shntaif, Saad Alghamdi, Ahd A. Mansour, Naeem F. Qusty, Azhar S. Sindi, Ahmad O. Babalghith, Ghazi A. Bamagous, Eman Adnan Abu-Seer

**Affiliations:** 1 Department of Pharmacy, Collage of Medicine and Health Science, Hajjah University, Hajjah, Yemen; 2 Department of Chemistry, College of Science for Women, University of Babylon, Alhilla, Iraq; 3 Laboratory Medicine Department, Faculty of Applied Medical Sciences, Umm Al-Qura University, Makkah, Saudi Arabia; 4 Medical Laboratory Science Department, Fakeeh College for Medical Sciences, Jeddah, Saudi Arabia; 5 Department of Clinical Laboratory Sciences, Faculty of Applied Medical Sciences, Umm Al-Qura University, Makkah, Saudi Arabia; 6 Medical genetics Department College of Medicine Umm alqura university, Makkah, Saudi Arabia; 7 Department of Pharmacology and Toxicology, Faculty of Medicine, Umm Al-Qura University, Makkah, Saudi Arabia; 8 Epidemiology department, Faculty of Public Health and Health informatics, Umm Al-Qura University, Makkah, Saudi Arabia; Vignan Pharmacy College, INDIA

## Abstract

This study aimed to design, synthesize, and evaluate novel morpholine-substituted phenoxyacetohydrazide derivatives as potential anti-inflammatory and anti-angiogenic agents. The compounds were synthesized and characterized by FTIR, NMR, mass spectrometry, and elemental analysis. In silico molecular docking revealed that compound **6e** exhibited strong binding affinities toward vascular endothelial growth factor (VEGF), cyclooxygenase-1 (COX-1), and cyclooxygenase-2 (COX-2), with docking scores of –13.1622, –12.5301, and –12.6705 (kcal/mol) respectively. The in vitro anti-inflammatory activity, assessed via the human red blood cell (HRBC) membrane stabilization assay, showed that compound 6e achieved an IC₅₀ value of 155 μg/mL, indicating potent efficacy. Additionally, **6e** demonstrated significant anti-angiogenic activity in both in vivo and ex vivo chick chorioallantoic membrane (CAM) models, inhibiting VEGF-induced angiogenesis in a dose-dependent manner and markedly reducing microvessel density and vessel length. In a rat model of alkali-induced corneal neovascularization, compound **6e** substantially suppressed neovascular growth. Moreover, in the carrageenan-induced paw edema model, it effectively reduced edema, neutrophil infiltration, and myeloperoxidase activity. Collectively, these findings position compound **6e** as a promising dual-action therapeutic candidate for treating chronic inflammation and pathological angiogenesis. This study underscores the potential of systematically designed phenoxyacetohydrazide scaffolds as multi-targeted therapeutic agents.

## Introduction

Inflammation is a vital physiological response that serves as a defense mechanism against harmful stimuli such as pathogens, tissue injury, and irritants. Characterized by symptoms like erythema, edema, increased temperature, and pain, inflammation plays a central role in the healing process [1–4]. However, when inflammation becomes chronic or poorly regulated, it may lead to a variety of pathological situations, including autoimmune diseases, cardiovascular diseases, and chronic inflammatory diseases such as rheumatoid arthritis and inflammatory bowel disease (IBD) [5]. The potential for inflammation to be both a protective response and a harmful one has made this process of great interest to biomedical research [6,7]. Contemporary research has elucidated the intricate cross-talk between inflammation and angiogenesis, which is defined as the formation of new blood vessels sprouting from pre-existing ones [8]. Under normal circumstances, angiogenesis is a tightly regulated process essential for growth, maturation, and wound healing [7,9,10]. In pathological conditions, however, dysregulated angiogenesis may exacerbate inflammatory responses and contribute to the progression of diseases [11–12]. In cancer, chronic inflammation is a hallmark of the tumor microenvironment; consequently, it stimulates angiogenesis for aggressive tumor growth and metastasis [7,13]. In diabetic retinopathy, as well as in cardiovascular diseases with ischemia, there are also findings of persistent inflammation concomitantly with aberrant vascular neovascularization [14]. The interface between the process of inflammation and angiogenesis is orchestrated by a lot of biochemical actors, including cytokines, growth factors, and components of the extracellular matrix [15].

Angiogenesis is associated with Chronic inflammation in various pathological conditions by blood vessel proliferation and enlargement and changes in vessel phenotype. Key pro-inflammatory cytokines—such as TNF-α and IL-1β—are known to induce endothelial proliferation and migration. Another link between inflammation and angiogenesis is provided Inflammatory mediators (e.g., prostaglandins from COX-2 activity) directly upregulate VEGF expression by the prostaglandin E series through COX-2 in several cell types. [16], fostering a microenvironment conducive to vascular proliferation. This dynamic crosstalk establishes a pathological feed-forward loop that amplifies disease severity and complicates therapeutic intervention [17–20]. The bidirectional nature of this relationship increases the pathophysiological complexity of diseases characterized by chronic inflammation, further emphasizing the therapeutic need for strategies that could effectively modulate both inflammatory processes. Morpholine (tetrahydro-1,4-oxazine) is heterocyclic containing nitrogen and oxygen. a versatile used in medicinal chemistry because of its favorable physicochemical characteristics, possible biological activity, and positive metabolic profile. Derivatives of morpholine have a significant contribution to make in the drug discovery program and are accountable for extensive research in varied biological activities. Surprisingly, the initial morpholine-derived drug to receive clinical approval was Preludin, which was introduced in 1955 as an anti-obesity agent illustrating the pharmacological versatility of this scaffold. This was followed by the development of morpholine analogues with significant analgesic activity. The synthesis of heterocyclic compounds containing multi-structure like that is pyridine and also phenoxy acetyl compounds in a molecule is receiving much attention in recent years to their wide range of biological activities. Several scientific literature data have indicated that phenoxyacetic acid and morpholine derivatives possess excellent anti-inflammatory and anticancer activities [21–33]. Moreover, phenoxyacetohydrazide derivatives have proven to be valuable leads, showing strong inhibition of tumor cell growth and modulation of important inflammatory signaling mechanisms [17,34,35]. The compounds explored in the present study feature critical pharmacophoric elements known to contribute to anti-inflammatory activity, including a hydrophobic pyridine ring, a hydrogen bond-donating NH group, an electron-rich nitrogen atom, and a distal phenoxy ring that may impart molecular recognition. In addition, the amide oxygen is speculated to form hydrogen bonding interactions with arginine residues within cyclooxygenase (COX) active sites, thus facilitating binding affinity. This functional group may also play a central role in augmenting COX selectivity and general anti-inflammatory activity.

In the present study, a molecular hybridization strategy was used to develop novel hybrids of phenoxyacetohydrazide by introducing benzyl and morpholine moieties to enhance their anti-angiogenic and anti-inflammatory activities. These modifications at the structural level were logically developed and implemented in a stepwise manner to enhance drug-like characters such as lipophilicity, receptor affinity, and pharmacokinetic profiles. Directed synthesis of morpholine-substituted compounds was driven by the predicted relationship between structural attributes and improved bioactivity*.* This research aimed to promote the creation of more potent therapeutic agents for diseases related to inflammation through the design of novel phenoxyacetohydrazide derivatives with dual anti-inflammatory and anti-angiogenic properties.

## Materials and methods

### Chemistry

The chemicals used in this study were obtained from Aldrich Chemical Co. Thin-layer chromatography (TLC) was carried out on silica plates backed with aluminum, and the spots were visualized under UV light. Melting points were determined using a Thomas Hoover capillary melting point apparatus equipped with a digital thermometer. Infrared (IR) spectra were recorded using the potassium bromide (KBr) pellet method on an FT-IR Shimadzu 8300 spectrophotometer. Nuclear magnetic resonance (NMR) spectra were measured on a Bruker 400 MHz NMR spectrophotometer in DMSO, with chemical shifts reported in parts per million (ppm) relative to tetramethylsilane as a reference. Mass spectra were obtained using a VG70-70H spectrophotometer, and major fragments are reported with their relative intensities in parentheses. Elemental composition results obtained through X-ray fluorescence (XRF) analysis were within 0.4% of theoretical values.

### General synthetic procedure for phenoxy acetic/butyric acid ethyl ester derivatives (3a–h)

The starting ester derivatives were synthesized by the reaction of (0.05 mol) substituted phenols **1a–h** with (0.075 mol) of substituted ester **2a–b** in (40 ml) dry acetone with (0.075 mol) anhydrous potassium carbonate. The reaction mixture was refluxed for 8–10 h. Then the reaction mixture was cooled and removed the solvent by distillation. To remove remaining potassium carbonate, the residual mass was triturated with cold water, and extracted with ether (3 × 30 ml). The organic layer was washed with NaOH solution (10%) (3 × 30 ml) followed by water (3 × 30 ml), dried over anhydrous sodium sulfate, and evaporated under reduced pressure to afford compounds **3a–h** [13].

#### (4-Chloro-phenoxy)-acetic acid ethyl ester (3a).

Yield 83%; FT-IR (cm^-1^): 1742 (C = O), 1281 (C-O-C); ^1^H NMR (CDCl_3_): σ 1.37 (t, 3H, CH_3_ of ester), 4.45 (q, 2H, CH_2 ester_), 4.98 (s, 2H, OCH_2_), 7.21 (d, J = 8.80 Hz, 2H, H_Ar_), 7.39 (d, J = 8.85 Hz, 2H, H_Ar_); LC–MS m/z 215 [M]^+^, 217 [M + 2]. Anal. Calcd. for C_10_H_11_BrO_3_: C, 46.36; H, 4.28. Found: C, 46.29; H, 4.32%.

#### (4-Bromo-phenoxy)-acetic acid ethyl ester (3b).

Yield 87%; FT-IR (cm^-1^): 1733 (C = O), 1287 (C-O-C); ^1^H NMR (CDCl_3_): σ 1.34 (t, 3H, CH_3_ of ester), 4.31 (q, 2H, CH_2ester_), 5.05 (s, 2H, OCH_2_), 6.83 (d, *J* = 8.80 Hz, 2H, H_Ar_), 7.35 (d, **J* *= 8.85 Hz, 2H, H_Ar_); LC–MS m/z 259 [M]^+^, 261 [M + 2]. Anal. Calcd. for C_10_H_11_BrO_3_: C, 46.36; H, 4.28. Found: C, 46.29; H, 4.32%.

#### O-tolyloxy-acetic acid ethyl ester (3c).

Yield 83%; FT-IR (cm^-1^): 1735 (C = O), 1279 (C-O-C); ^1^H NMR (CDCl3): σ 1.35 (t, 3H, CH_3ester_), 2.16 (s, 3H, CH_3_), 4.31 (q, 2H, CH_2 ester_), 5.01 (s, 2H, CH_2_), 6.82 (d, *J* = 8.80 Hz, 1H, H_Ar_), 7.37 (d, *J* = 7.40 Hz, 1H, H_Ar_), 7.54 (t, *J* = 8.80 Hz, 2H, H_Ar_), LC–MS m/z 195 [M]^+^. Anal. Calcd. for C_11_H_14_O_3_: C, 68.02; H, 7.27. Found: C, 68.14; H, 7.16%.

#### (2,6-Dimethyl-phenoxy)-acetic acid ethyl ester (3d).

Yield 89%; FT-IR (cm^-1^): 1735 (C = O), 1284 (C-O-C); ^1^H NMR (CDCl_3_): σ 1.31 (t, 3H, CH3 of ester), 1.31 (s, 6H, 2CH_3_), 4.27 (q, 2H, CH_2ester_), 5.02 (s, 2H, OCH_2_), 6.92-7.23 (m, 3H, H_Ar_); LC–MS m/z 209 [M + 1]. Anal. Calcd. for C_12_H_16_O_3_: C, 69.21; H, 7.74. Found: C, 69.18; H, 7.71%.

#### 4-(4-Fluoro-phenoxy)-butyric acid ethyl ester (3e).

Yield 85%; FT-IR (cm^-1^): 1735 (C = O), 1284 (C-O-C); ^1^H NMR (CDCl_3_): σ 1.31

(t, 3H, CH_3ester_), 2.21 (m, 2H, CH_2_), 2.75 (t, 2H, COCH_2_), 4.11 (t, 2H, OCH_2_), 4.27 (q, 2H, CH_2 ester_), 6.92 (d, *J* = 8.80 Hz, 2H, H_Ar_), 7.59 (d, *J* = 8.85 Hz, 2H, H_Ar_); LC–MS m/z 199 [M]^+^, 201 [M + 2]. Anal. Calcd. for C_12_H_15_FO_3_: C, 63.70; H, 6.68. Found: C, 63.57; H, 6.62%.

#### (2,4-Diisopropyl-phenoxy)-acetic acid ethyl ester (3f).

Yield 89%; FT-IR (cm^-1^): 1735 (C = O), 1284 (C-O-C); ^1^H NMR (CDCl_3_): σ 1.16 (s, 6H, 2CH_3_), 1.26 (s, 6H, 2CH_3_), 1.32 (t, 3H, CH_3ester_), 3.10 (s, 2H, 2CH), 4.27 (q, 2H, CH_2ester_), 5.02 (s, 2H, OCH_2_), 6.87-7.39 (m, 3H, H_Ar_); LC–MS m/z 265 [M + 1]. Anal. Calcd. for C_16_H_24_O_3_ C, 72.69; H, 9.15. Found: C, 72.66; H, 9.12%.

#### 4-(4-Bromo-phenoxy)-butyric acid ethyl ester (3g).

Yield 81%; FT-IR (cm^-1^): 1733 (C = O), 1287 (C-O-C); ^1^H NMR (CDCl_3_): σ 1.34 (t, 3H, CH_3 ester_), 2.26 (m, 2H, CH_2_), 2.72 (t, 2H, COCH_2_), 4.05 (t, 2H, OCH_2_), 4.31 (q, 2H, CH_2ester_), 6.83 (d, **J* *= 8.80 Hz, 2H, H_Ar_), 7.35 (d, **J* *= 8.85 Hz, 2H, H_Ar_); LC–MS m/z 288 [M + 1]. Anal. Calcd. for C_12_H_15_BrO_3_: C, 50.19; H, 5.27. Found: C, 50.29; H, 5.18%.

#### 4-(3-Nitro-phenoxy)-butyric acid ethyl ester (3h).

Yield 82%; FT-IR (cm^-1^): 1742 (C = O), 1284 (C-O-C); 1H NMR (CDCl_3_): σ 1.34 (t, 3H, CH_3_ ester), 2.23 (m, 2H, CH_2_), 2.73 (t, 2H, COCH_2_), 4.05 (t, 2H, OCH_2_), 4.29 (q, 2H, CH_2ester_), 6.84 (d, *J* = 7.20 Hz, 2H, H_Ar_), 7.45 (s, 1H, H_Ar_), 7.62 (t, **J* *= 8.80 Hz, 1H, H_Ar_); LC–MS m/z 254 [M + 1]. Anal. Calcd. for C_12_H_15_NO_5_: C, 56.91; H, 5.97. Found: C, 56.80; H, 5.91%.

### General synthetic procedure for phenoxy-acetic acid/butyric acid hydrazide derivatives (4a–h)

To the solution of (0.03 mol) ester analogs **3a–h** in (20 ml) ethanol, (0.045 mol) hydrazine hydrate was added, the reaction mixture was stirred in RT for 7 h. Reaction complication was monitored by TLC using the mixture (hexane:ethylacetate 2:1). The reaction mixture was allowed to stand overnight. The white precipitates (**4a-h**) were formed which were separated by filtration, washed and dried. The products recrystallized from ethanol [12,13,17].

#### (4-Chloro-phenoxy)-acetic acid hydrazide (4a).

Yield 89%; mp 116−118 °C; FT-IR (KBr, cm^-1^): 3298 (NH_2_), 3303 (NH), 1701 (C = O); ^1^H NMR (CDCl_3_): σ 3.57 (s, 2H, NH_2_), 4.92 (s, 2H, OCH_2_), 6.88 (d, *J* = 8.80 Hz, 2H, H_Ar_), 6.88 (d, *J* = 8.85 Hz, 2H, H_Ar_), 8.22 (t,1H, NH), LC–MS m/z 201 [M]^+^, 203 [M + 2]. Anal. Calcd. for C_8_H_9_ClN_2_O_2_: C, 47.89; H, 4.52; N, 13.96. Found: C, 47.78; H, 4.56; N, 14.08%.

#### (4-Bromo-phenoxy)-acetic acid hydrazide (4b).

Yield 91%; mp 119–120 °C; FT-IR (KBr, cm^-1^): 3345 (NH_2_), 3203 (NH), 1652 (C = O); ^1^H NMR (CDCl_3_): σ 3.77 (s, 2H, NH_2_), 4.95 (s, 2H, OCH_2_), 6.74 (d, **J* *= 8.80 Hz, 2H, H_Ar_), 7.11 (d, *J* = 8.85 Hz, 2H, H_Ar_), 8.50 (t, 1H, NH); LC–MS m/z 245 [M]^+^, 247 [M + 2]. Anal. Calcd. For C_8_H_9_BrN_2_O_2_: C, 39.21; H, 3.70; N, 11.43. Found: C, 39.30; H, 3.74; N, 11.55%.

#### O-tolyloxy-acetic acid hydrazide (4c).

Yield 79%; mp 116-118^o^C; FT-IR (KBr, cm^−1^): 3401 (NH_2_), 3311 (NH),1684 (C = O); ^1^H-NMR (CDCl_3_): δ 2.16 (s, 3H, CH_3_), 3.83 (d, 2H, NH_2_), 5.03 (s, 2H, OCH_2_), 6.82 (d, **J* *= 8.80 Hz, 1H, H_Ar_), 7.37 (d, **J* *= 7.40 Hz, 1H, H_Ar_), 7.48 (t, **J* *= 8.80 Hz, 2H, H_Ar_), 8.33 (t, 1H, NH); LC-MS m/z 181 [M + 1]. Anal. Calcd. for C_9_H_12_N_2_O_2_: C, 59.99; H, 6.71; N, 15.55. Found: C, 60.07; H, 6.65; N, 15.62%.

#### 2-(2,4-Dimethylphenoxy)acetohydrazide (4d).

Yield 85%; mp 111–113 °C; FT-IR (KBr, cm^-1^): 3311 (NH_2_), 3213 (NH), 1670 (C = O); ^1^H NMR (CDCl_3_): σ 1.31 (s, 6H, 2CH_3_), 3.87 (d, 2H, NH_2_), 5.05 (s, 2H, OCH_2_), 6.92-7.45 (m, 3H, H_Ar_), 8.42 (t, 1H, NH); LC–MS m/z 195 [M + 1]. Anal. Calcd. for C_10_H_14_N_2_O_2_: C, 61.84; H, 7.27; N, 14.42. Found: C, 61.81; H, 7.23; N, 14.39%.

#### 4-(4-Fluoro-phenoxy)-butyric acid hydrazide (4e).

Yield 93%; mp 82–84 °C; FT-IR (KBr, cm^-1^): 3323 (NH_2_), 3209 (NH), 1682 (C = O); ^1^H NMR (CDCl_3_): σ 3.01 (m, 2H, CH_2_), 2.81 (t, 2H, COCH_2_), 3.90 (s, 2H, NH_2_), 4.09 (t, 2H, OCH_2_), 7.02 (d, J = 8.80 Hz, 2H, H_Ar_), 7.44 (d, J = 8.85 Hz, 2H, H_Ar_), 8.49 (s, 1H, NH); LC–MS m/z 213 [M + 1]. Anal. Calcd. for C_10_H_13_FN_2_O_2_: C, 56.60; H, 6.17; N, 13.20. Found: C, 56.62; H, 6.22; N, 13.24%.

#### 2-(2, 4-Diisopropylphenoxy)acetohydrazide (4f).

Yield 85%; mp 111–113 °C; FT-IR (KBr, cm^-1^): 3311 (NH_2_), 3213 (NH), 1670 (C = O); ^1^H NMR (CDCl3): σ 1.16 (s, 6H, 2CH_3_), 1.26 (s, 6H, 2CH_3_), 1.32 (t, 3H, CH_3ester_), 3.10 (s, 2H, 2CH), 3.87 (d, 2H, NH_2_), 5.05 (s, 2H, OCH_2_), 6.92-7.59 (m, 3H, H_Ar_), 8.42 (t, 1H, NH); LC–MS m/z 251 [M + 1]. Anal. Calcd. for C_14_H_22_N_2_O_2_: C, 67.17; H, 8.86; N, 11.19. Found: C, 67.15; H, 8.82; N, 11.15%.

#### 4-(4-Bromo-phenoxy)-butyric acid hydrazide (4g).

Yield 78%; mp 87–88 °C; FT-IR (KBr, cm^-1^): 3330 (NH_2_), 3247 (NH), 1677 (C = O); 1H NMR (CDCl_3_): σ 2.26 (m, 2H, CH_2_), 2.72 (t, 2H, COCH_2_), 3.85 (s, 2H, NH_2_), 4.07 (t, 2H, OCH_2_), 6.83 (d, *J* = 8.80 Hz, 2H, H_Ar_), 7.35 (d, *J* = 8.85 Hz, 2H, H_Ar_), 8.40 (s, 1H, NH); LC–MS m/z 273 [M}+ , 275 [M + 2]. Anal. Calcd. for C_10_H_13_BrN_2_O_2_: C, 43.97; H, 4.80; N, 10.26. Found: C, 43.84; H, 4.87; N, 10.28%.

#### 4-(3-Nitro-phenoxy)-butyric acid hydrazide (4h).

Yield 76%; mp 92–94 °C; FT-IR (KBr, cm^-1^): 3311 (NH_2_), 3265 (NH), 1659 (C = O); ^1^H NMR (CDCl_3_): σ 2.31 (m, 2H, CH_2_), 2.80 (t, 2H, COCH_2_), 3.97 (s, 2H, NH_2_), 3.98 (t, 2H, OCH_2_), 6.73 (d, *J* = 7.20 Hz, 2H, H_Ar_), 7.60 (s, 1H, H_Ar_), 7.62 (t, *J* = 8.80 Hz, 1H, H_Ar_), 8.46 (t, 1H, NH); LC–MS m/z 240 [M + 1]. Anal. Calcd. for C_10_H_13_N_3_O_4_: C, 50.21; H, 5.48; N, 17.56. Found: C, 50.23; H, 5.35; N, 17.62%.

### General synthetic procedure for phenyl 2-(4-benzylmorpholine-2-carbonyl) hydrazine-1-carboxylate (6e-h)

A solution comprising (2 mmol) of phenoxy-acetic acid/butyric acid hydrazide derivatives (4a–m) along with (30 ml) dry DCM at 25–30 °C, was treated with (3 mmol) lutidine, followed by the addition of (2 mmol) of 4-benzylmorpholine-2-carboxylic acid. The resulting mixture was then stirred for 30 min, then cooled to 0–5 °C and TBTU (2 mmol) was added drop wise for 30 min with kept the temperature below 5 °C. The reaction mass kept overnight with constant stirring was accomplished and monitored by TLC using the mixture (ethyl acetate:Hexane 4:1). The solvent was evaporated under reduced pressure, after added crushed ice, the resulting precipitate was filtered, dried, and subsequently crystallized from ethanol to afford compounds **6a–h** in good yield [12].

#### 4-Benzyl-N’-(2-(4-chlorophenoxy)acetyl)morpholine-2-carbohydrazide (6a).

Yield 72%; M.P112 - 114 °C; FT-IR (KBr, cm^-1^): 1650 (C = O), 1678 (amide, C = O), 3275−3360 (NH-NH); ^1^H NMR (DMSO-d6): δ 2.68 (t, 4H, **J* *= 8.0 Hz, NCH_2morpholine ring_), 3.57 (s, 2H, NCH_2_), 3.57 (t, **J* *= 7.80 Hz, 1H, CH_morpholine ring_), 4.09 (t, **J* *= 7.80 Hz, 2H, OCH_2 morpholine ring_), 5.20 (s, 2H, OCH_2_), 7.05-7.39 (m, 9H, H_Ar_), 9.75 (bs, 1H, NH), 9.91(bs, 1H, NH); 13C NMR (DMSO-d6): δ 55.76, 59.70, 64.70, 90.10, 117.41 127.11, 128.80, 130.91, 138.60, 156.22, 166.30, 169.90; LC-MS m/z 404 [M]+ , 406 [M + 2]. Anal. Calcd. for C_20_H_22_ClN_3_O_4_: C, 59.48; H, 5.49; Cl, 8.78; N, 10.40, Found: C, 59.50; H, 5.52; Cl, 8.80; N, 10.42%.

#### 4-Benzyl-N’-(2-(4-bromophenoxy)acetyl)morpholine-2-carbohydrazide (6b).

Yield 82%; M.P 124–126 °C; FT-IR (KBr, cm^-1^): 1650 (C = O), 1678 (C = O)_amide_, 3275−3360 (NH-NH); ^1^H NNMR (DMSO-d6): δ 2.68 (t, 4H, **J* *= 8.0 Hz, NCH_2 morpholine ring_), 3.57 (s, 2H, NCH_2_), 3.57 (t, **J* *= 7.80 Hz, 1H, CH_morpholine ring_), 4.09 (t, **J* *= 7.80 Hz, 2H, OCH_2morpholine ring_), 5.20 (s, 2H, OCH_2_), 7.05-7.39 (m, 9H, H_Ar_), 9.75 (bs, 1H, NH), 9.91(bs, 1H, NH); ^13^CNMR (DMSO-*d6*): δ 55.76, 59.70, 64.70, 90.10, 117.41 127.11, 128.80, 130.91, 138.60, 156.22, 166.30, 169.90; LC-MS m/z 448 [M]+ , 450 (M + 2). Anal. Calcd. for C_20_H_22_BrN_3_O_4_: C, 53.58; H, 4.95; Br, 17.82; N, 9.37, Found: C, 53.60; H, 4.97; Br, 17.84; N, 9.39%.

#### 4-Benzyl- N’-(2-(o-tolyloxy)acetyl)morpholine-2-carbohydrazide (6c).

Yield 77%; M.P 160–162 °C; FT-IR (KBr, cm^-1^): 1650 (C = O), 1678 (amide, C = O), 3275–3360 (NH-NH); ^1^H NMR (DMSO-d6): δ 2.60 (s, 3H, CH3), 2.68 (t, 4H, **J* *= 8.0 Hz, NCH_2morpholine ring_), 3.57 (s, 2H, NCH_2_), 3.57 (t, **J* *= 7.80 Hz, 1H, CH _morpholine ring_), 4.09 (t, **J* *= 7.80 Hz, 2H, OCH_2 morpholine ring_), 5.20 (s, 2H, OCH_2_), 6.85-7.29 (m, 9H, H_Ar_), 9.75 (bs, 1H, NH), 9.91(bs, 1H, NH); ^13^CNMR (DMSO-d6): δ 15.41, 55.76, 59.70, 64.70, 90.10, 117.41 127.11, 128.80, 130.91, 138.60, 156.22, 166.30, 169.90; LC-MS m/z 383 [M]+ , 384 [M + 1]. Anal. Calcd. for C_21_H_25_N_3_O_4_: C, 65.78; H, 6.57; N, 10.96, Found: C, 65.80; H, 6.59; N, 10.98%.

#### 4-Benzyl-N’-(2-(2,4-dimethylphenoxy)acetyl)morpholine-2-carbohydrazide (6d).

Yield 84%; M.P 110–112 °C; FT-IR (KBr, cm^-1^): 1650 (C = O), 1678 (C = O)_amide_, 3275–3360 (NH-NH); ^1^H NMR (DMSO-d6): δ 2.14 (s, 6H, CH_3_), 2.68 (t, 4H, **J* *= 8.0 Hz, NCH_2morpholine ring_), 3.57 (s, 2H, NCH_2_), 3.57 (t, **J* *= 7.80 Hz, 1H, CH_morpholine ring_), 4.09 (t, **J* *= 7.80 Hz, 2H, OCH_2 morpholine ring_), 5.20 (s, 2H, OCH_2_), 6.66-7.29 (m, 8H, H_Ar_), 9.75 (bs, 1H, NH), 9.91(bs, 1H, NH); ^13^CNMR (DMSO-d6): δ 15.71, 21.66, 55.76, 59.70, 64.70, 90.10, 117.41 127.11, 128.80, 130.91, 138.60, 156.22, 166.30, 169.90; LC-MS m/z 397 [M]^+^, 398 [M + 1]. Anal. Calcd. for C_22_H_27_N_3_O_4_: C, 66.48; H, 6.85; N, 10.57, Found: C, 66.50; H, 6.87; N, 10.59%.

#### 4-Benzyl-N’-(4-(4-fluorophenoxy)butanoyl)morpholine-2-carbohydrazide (6e).

Yield 91%; M.P 115–117 °C; FT-IR (KBr, cm^-1^): 1650 (C = O), 1678 (C = O)_amide_, 3275−3360 (NH-NH); ^1^H NMR (DMSO-d6): δ 2.06 (m, 4H, CH_2_), 2.88 (t, 4H, **J* *= 8.0 Hz, NCH_2 morpholine ring_), 3.57 (s, 4H, NCH_2_), 4.11 (s, **J* *= 7.80 Hz, 2H, OCH_2_), 4.62 (t, **J* *= 7.80 Hz, 1H, CH _morpholine ring_), 7.09-7.29 (m, 9H, H_Ar_), 10.15 (bs, 1H, NH), 10.21(bs, 1H, NH); ^13^CNMR (DMSO-d6): δ 25.23, 34.51, 55.76, 59.70, 64.70, 68.66, 90.10, 116.11 128.11, 138.60, 155.02, 169.90, 176.66; LC-MS m/z 404 [M]^+^, 406 [M + 2]. Anal. Calcd. for C_22_H_26_FN_3_O_4_: C, 63.60; H, 6.31; F, 4.57; N, 10.11, Found: C, 63.62; H, 6.33; F, 4.59; N, 10.13%.

#### 4-Benzyl-N’-(2-(2,4-diisopropylphenoxy)acetyl)morpholine-2-carbohydrazide (6f).

Yield 83%; M.P 244–246 °C; FT-IR (KBr, cm^-1^): 1650 (C = O), 1678 (C = O)_amide_, 3275–3360 (NH-NH); ^1^H NMR (DMSO-d6): δ 1.14-1.20 (m, 12H, CH_3_), 2.68 (t, 4H, **J* *= 8.0 Hz, NCH_2morpholine ring_), 2.87 (s, 1H, CH_3_), 3.05 (s, 1H, CH_3_), 3.63 (s, 2H, NCH_2_), 4.03 (t, **J* *= 7.80 Hz, 1H, CH_morpholine ring_), 4.69 (t, **J* *= 7.80 Hz, 2H, OCH_2morpholine ring_), 5.20 (s, 2H, OCH2), 6.66-7.29 (m, 8H, H_Ar_), 9.75 (bs, 1H, NH), 9.91(bs, 1H, NH); ^13^CNMR (DMSO-d6): δ 23.66, 27.65.33.52, 55.76, 59.70, 64.70,66.61, 90.10, 111.21, 124.31, 127.20, 128.43, 138.60, 140.44, 152.92, 166.30, 169.90; LC-MS m/z 455 [M]^+^. Anal. Calcd. for C_26_H_35_N_3_O_4_C, 68.85; H, 7.78; N, 9.26, Found: C, 68.87; H, 7.80; N, 9.28%.

#### 4-Benzyl-N’-(4-(4-bromophenoxy)butanoyl)morpholine-2-carbohydrazide (6g).

Yield 77%; M.P 117–119 °C; FT-IR (KBr, cm^-1^): 1650 (C = O), 1678 (C = O)_amide_, 3275–3360 (NH-NH); ^1^H NMR (DMSO-d6): δ 2.21 (m, 4H, CH_2_), 2.84 (t, 4H, **J* *= 8.0 Hz, NCH_2morpholine ring_), 3.67 (s, 4H, NCH_2_), 4.11 (s, **J* *= 7.80 Hz, 2H, OCH_2_), 4.62 (t, **J* *= 7.80 Hz, 1H, CH _morpholine ring_), 6.98-7.48 (m, 9H, H_Ar_), 10.15 (bs, 1H, NH), 10.21(bs, 1H, NH); ^13^CNMR (DMSO-d6): δ 14.88, 20.42, 55.86, 64.70, 98.01, 114.71, 118.44, 128.11, 132.32, 138.61, 158.42, 169.90, 200.01; LC-MS m/z 477 [M]+ , 479 [M + 2]. Anal. Calcd. for C_22_H_26_BrN_3_O: C, 55.47; H, 5.50; Br, 16.77; N, 8.82, Found: C, 55.49; H, 5.52; Br, 16.79; N, 8.84%.

#### 4-Benzyl-N’-(4-(3-nitrophenoxy)butanoyl)morpholine-2-carbohydrazide (6h).

Yield 76%; M.P 199–201 °C; FT-IR (KBr, cm^-1^): 1650 (C = O), 1678 (C = O)_amide_, 3275−3360 (NH-NH); ^1^H NMR (DMSO-d6): δ 2.21 (m, 4H, CH2), 2.84 (t, 4H, **J* *= 8.0 Hz, NCH_2morpholine ring_), 3.67 (s, 4H, NCH_2_), 4.11 (s, **J* *= 7.80 Hz, 2H, OCH_2_), 4.62 (t, **J* *= 7.80 Hz, 1H, CH_morpholine ring_), 7.21-7.91 (m, 9H, H_Ar_), 10.15 (bs, 1H, NH), 10.21(bs, 1H, NH); ^13^CNMR (DMSO-d6): δ 25.22, 34.51, 55.86, 59.62, 64.70, 68.61, 90.01, 111.71, 115.54, 128.81, 130.32, 151.42, 158.31, 169.90, 176.25c; LC-MS m/z 443 [M]^+^.Anal. Calcd. for C_22_H_26_N_4_O_6_: C, 59.72; H, 5.92; N, 12.66, Found: C, 59.74; H, 5.94; N, 12.68%.

### ADMET

In the current study, we assessed the ADMET (Adsorption, Distribution, Metabolism, Excretion, and Toxicity) properties of each of the four selected molecules using the Swiss ADME (http://www.swissadme.ch) and Admet “SAR” (http://lmmd.ecust.edu.cn/admetsar2/) online tools, which are used to predict vital pharmaceutical and toxicological properties. The physicochemical properties of phenoxyacetohydrazide derivatives, such as canonical SMILES, formula, molecular weight, number of heavy atoms, aromatic heavy atoms, rotatable bonds, H-bond acceptors, H-bond donors, etc. were collected from web-based online servers such as Swiss ADME. Caco-2 cell permeability, brain/blood barrier, human intestinal absorption, carcinogens and acute oral toxicity were calculated using ADMET Lab.

### Molecular docking analysis

The *in silico* molecular docking study was carried out to assess the binding interactions of compound **6e** with three key target proteins: vascular endothelial growth factor (VEGF, PDB ID: 1RV6), cyclooxygenase-1 (COX-1, PDB ID: 1EQG), and cyclooxygenase-2 (COX-2, PDB ID: 1CVU). The crystal structures of these proteins were obtained from the Protein Data Bank (PDB). Indomethacin served as our standard comparator due to its known mechanism of action against Cyclooxygenase (COX) enzymes, making it an effective treatment as anti-inflammatory moreover, the Nonsteroidal anti-inflammatory drugs (NSAIDs) as Indomethacin inhibit angiogenesis by inhibition vascular endothelial growth factor (VEGF) [36]. The docked poses of compound **6e** within the binding pockets of VEGF, COX-1, and COX-2 were visualized London dG scoring function within MOE to evaluate the binding affinity of compound **6e** to these proteins. The 3D representations, 2D schematic diagrams, and ball-and-stick models were generated to illustrate the binding interactions and the spatial arrangement of the compound within the protein binding sites. To validate the reliability of the docking protocol, redocking of the native co-crystallized ligands was performed for each target protein (VEGF: PDB ID 1RV6, COX-1: PDB ID 1EQG, COX-2: PDB ID 1CVU). The Root-Mean-Square Deviation (RMSD) values between the redocked and crystallographic poses were calculated. An RMSD value of less than 2.0 Å was achieved for all targets (VEGF (PDB: 1RV6): 1.25 Å, COX-1 (PDB: 1EQG): 1.42 Å and COX-2 (PDB: 1CVU): 1.18 Å), confirming the accuracy and reliability of the docking methodology.

### Protein preparation

Pre-processing of the protein structures was performed with the Molecular Operating Environment (MOE) software. Protein structures underwent standard bioinformatics pre-processing, which included the It involved adding missing hydrogen atoms and incomplete residues since these components are very essential in maintaining the protein chain’s structural integrity. Further, specific protonation states were assigned, considering a physiological pH of 7.2, thus enabling more realistic modelling of interactions. A crucial part of the preparation was the removal of all heteroatoms, which could interfere with the docking simulations. The energy minimization and geometry optimization using the CHARMM force field was indispensable. The smart minimizer technique, which combines steepest descent and conjugate gradient algorithms, ensured that the potential energy of the system was reduced while keeping the proteins structurally stable deletion of crystallographic water molecules and energy minimization to yield stable conformations acceptable for docking simulations. Compound **6e** was similarly prepared by conformational optimization and appropriate protonation state adjustment before docking [37,38].

### Ligand preparation

The two-dimensional structure of compound 6e was built using ACD/ChemSketch and minimize its free energy of the of ligand was done in Steepest Descent and Conjugate Gradient methods using Discovery Studio 2024 Client Software Inc.) The minimized structure was imported into the MOE software. The two-dimensional structure was then converted to a three-dimensional model, and appropriate protonation states at physiological pH were assigned.

## Biology

### Human red blood cells (HRBC) membrane stabilization assay

The human red blood cell (HRBC) membrane stabilization method was employed to evaluate the anti-inflammatory activity of the compounds, as previously described [17,39,40]. For test the *in vitro* anti-inflammatory activity of synthesized compounds (**6e-h**), blood samples were collected from healthy volunteers who had not taken NSAIDs for 14 days. The blood was mixed with an equal volume of sterilized Alsever’s solution, centrifuged at 3000 rpm, and the packed cells were washed with isotonic saline (0.9% w/v NaCl). A 10% cell suspension was then prepared using isotonic saline. The test compounds were dissolved in DMSO to prepare solutions at concentrations of 50, 100, 150, 200, and 250 μg/ml. For each concentration, a mixture containing 1 ml of phosphate buffer, 2 ml of hypotonic saline, and 0.5 ml of the HRBC suspension was prepared. The samples were incubated at 37°C for 30 minutes and centrifuged at 3000 rpm for 20 minutes. The hemoglobin content in the supernatant was determined spectrophotometrically at 560 nm. Indomethacin at the same concentrations (50–250 μg/ml) was used as the reference standard, and a control sample was prepared without the test compounds. The percentage hemolysis was calculated by considering the hemolysis in the control group as 100%. The percentage of HRBC membrane stabilization or protection was determined using the following formula:


𝐩𝐞𝐫𝐜𝐞𝐧𝐭 𝐩𝐫𝐨𝐭𝐞𝐜𝐭𝐢𝐨𝐧=100−((𝐎𝐃 𝐨𝐟 𝐝𝐫𝐮𝐠 𝐭𝐫𝐞𝐚𝐭𝐞𝐝 𝐬𝐚𝐦𝐩𝐥𝐞/𝐎𝐃 𝐨𝐟 𝐜𝐨𝐧𝐭𝐫𝐨𝐥) ×100).

### *In vivo* chorioallontoic membrane (CAM) assay

The *in vivo* CAM assay was performed according to the standard protocol [7]. All the eggs were wiped with 75% alcohol prior to incubation. Then the fertilized eggs were incubated at 37°C with 60% relative humidity. On the 11th day of incubation, all the eggs divided into three treatment groups contain minimum 6 eggs as mentioned below.

Group I – Normal [PBS (Phosphate buffer saline) (10 mM Sodium phosphate mono basic+ 1.8 mM Sodium phosphate dibasic+140 mM sodium chloride, pH 7.4) alone]
Group II – Control [1µg of rVEGF165 alone]
Group III – 5 µg of **6e** + 1 µg of rVEGF165.

A rectangular window was made in the egg shell and Whatman filter paper (3 mm diameter) containing with or without compound **6e** (5 µg) was placed on the CAM and the window were sealed using sterile vegetable wrap. Windows were opened after 48h, i.e., on 13th day of incubation, observed by two independent observers and measured for change in the Micro Vessel Density (MVD) in the area beneath the cover slip and photographed using Sony steady shot DSC-W610 camera.

## Animals and ethics

### Animals and ethical approval.

Swiss albino female rats, matured by 150−160 g were housed under standard research Center which to encourage the eating routine of animal food and water which is not obligatory in the complete analysis. The Rats were kept up at the room temperature (27 °C ± 2oC) with excellent ventilation for a 12 h day/night cycle. Swiss albino rats (150 gm. Each) were utilized for the current examination. They were encouraged with a standard pellet diet and for which water was not obligatory. All the animals were acclimatized for things like a multi-week identity before the trial session. All animal experimentation was endorsed by the Institutional Animal Ethics Committee (IAEC), (Approval ID: HU-PHARM-ETHICS-2024–001). College of Medicine and Health Sciences, Hajjah University, Yemen. Efforts were made to minimize animal suffering, including the use of ketamine/xylazine anesthesia for invasive procedures. Humane endpoints were applied, and animals were euthanized by sodium pentobarbital (≥100 mg/kg, intraperitoneally) followed by cervical dislocation, as per ethical guidelines.

## Determination of the Lethal Dose 50 (LD50) of the 6e-h

The acute lethal dose 50 (LD50) toxicity test is performed in rodents as part of the safety assessment of many substances. The LD50 (median lethal dose) test is usually the first test conducted for every chemical before further toxicity tests are carried out. It is used for estimating the potential hazards of chemicals on humans [41]. Although its major endpoint is death, non-lethal acute effect may occur as signs of toxicity depending on the chemical being tested. The LD50 values were determined using the “staircase” method, as described in previous studies [11,13]. Male albino rats were randomly assigned to twelve groups, by use simple randomization, animals are randomly assigned (by chance) to generated similar numbers of experimental group. each consisting of six animals. Acute oral toxicity was evaluated following the OECD Guideline 425 (Up-and-Down Procedure), which is designed to minimize animal use while determining a compound’s toxicity range. The test ligand was administered orally at incremental doses of 1000, 2000, 3000, 4000, and 5000 mg/kg of body weight. Following administration, the animals were observed continuously for three hours to monitor general behavioral, neurological, and autonomic profiles. Subsequently, observations were made every 30 minutes for an additional three hours and, finally, mortality was recorded after 24 hours. The maximum non-lethal dose and the minimum lethal dose were established based on these observations. Based on these outcomes, therapeutic doses were selected as one-tenth (1/10) and one-twentieth (1/20) of the MLD, corresponding to 400 mg/kg and 200 mg/kg, respectively.

### Induction of alkali burn-induced corneal neovascularization.

Corneal neovascularization (CNV) was induced through alkali injury following a standard protocol with minor modifications [13]. Swiss albino rats were first examined to ensure the absence of ophthalmic diseases before the alkali burn procedure. The animals were divided into three groups, each comprising six animals (n = 6 per group):

**Group I**: Normal, treated with phosphate-buffered saline (PBS).
**Group II**: Control group, treated with 1 µg of recombinant VEGF (rVEGF).
**Group III**: Treated group, receiving rVEGF165 (10 µg) and compound **6e** (5 µg/ml).

An alkali burn was induced in the right eye of animals in Groups II and III after administering general anesthesia via intraperitoneal injection of ketamine (1 ml/kg) and chlorpromazine (1 ml/kg). A 3-mm diameter piece of Whatman filter paper soaked in 4 ml of 1 mol/L NaOH was applied to the center of the cornea for 30 seconds, followed by irrigation of the treated cornea with 60 ml of normal saline.

Group II animals were subsequently treated with 1 µg of rVEGF applied to the right eye. The extent of CNV was quantified through photographic documentation 48 hours after the alkali burn and rVEGF treatment. For Group III animals, the alkali-burned cornea was treated with compound **6e** (5 µg) alongside rVEGF165 (10 µg). The inhibitory effect of compound **6e** on neovascularization was evaluated through photographic documentation 24 hours after treatment with compound **6e**.

## Carrageenan-induced Paw Edema in rat

Female Swiss albino rats were selected for the study to evaluate the anti-inflammatory effects of compound **6e** against carrageenan-induced paw edema [25]. The animals were divided randomly into four groups, with each group consisting of six experimental animals (n = 6):

**Group 1**: Normal animals (untreated).
**Group 2**: Control group (saline-treated).
**Group 3**: Positive control group (indomethacin-treated).
**Group 4**: Animals treated with compound **6e**.

Prior to the experiment, animals in Group 2 were administered saline (5 mL/kg), animals in Group 3 received the positive control indomethacin (10 mg/kg), and animals in Group 4 were treated with compound **6e** (25 mg/kg). All animals were left undisturbed in their respective cages for 2 hours.

After this period, animals in the 2, 3 and 4 groups were in intraperitoneal with 100 µl of 2% carrageenan into the right hind paw to induce edema. The right paws of all experimental animals were then observed at different time intervals (2, 3 and 5 hours post-carrageenan injection), and the paw volume was measured using a digital plethysmometer to assess the degree of edema.

## Tissue myeloperoxidase activity (MPO) assays

Rats were treated either with or without positive control (indomethacin at 10 mg/kg body weight) or compound **6e** (25 mg/kg body weight) 2 hours prior to the injection of carrageenan into the right hind paw. The activity of tissue myeloperoxidase (MPO) was assessed 4 hours post-carrageenan injection [42,43]. Tissue samples were collected and placed in 0.75 ml of 80 mM phosphate-buffered saline (PBS, pH 5.4) containing 0.5% hexadecyltrimethylammonium bromide (HTAB). The samples were homogenized for 45 seconds at 0°C using a homogenizer. The homogenate was transferred into microfuge tubes, and the homogenizer vessel was rinsed with an additional 0.75 ml of HTAB in PBS. The wash was combined with the homogenate to yield a total sample volume of 1.5 ml, which was then centrifuged at 12,000 × g at 4°C for 15 minutes. For the MPO assay, 30 μl of the resulting supernatant was added to 96-well microtiter plates. To each well, 200 μl of a reaction mixture was added, consisting of 100 μl of 80 mM PBS (pH 5.4), 85 μl of 0.22 M PBS (pH 5.4), and 15 μl of 0.017% hydrogen peroxide. The reaction was initiated by adding 20 μl of 18.4 mM tetramethylbenzidine HCl dissolved in dimethylformamide. The plates were incubated at 37°C for 3 minutes, and the reaction was terminated by adding 30 μl of 1.46 M sodium acetate (pH 3.0). Enzyme activity was determined spectrophotometrically by measuring absorbance at 630 nm using a plate reader. Results were expressed as MPO activity per milligram of tissue (MOD/mg tissue). The assay was validated with appropriate positive controls (purified MPO enzyme) and negative controls (reagents without tissue sample). All samples were measured in six times (n = 6 technical replicates) for statistical accuracy.

###   Results and discussion Structure-activity relationship of 4-Benzyl-N’-(4-(4 fluorophenoxy)butanoyl)morpholine-2-carbohydrazide

The morpholine ring provides a rigid, heterocyclic scaffold that likely contributes to the compound’s overall pharmacokinetic properties, such as absorption, distribution, metabolism, and excretion (ADME). Its conformational rigidity might be important for optimal interaction with the target protein(s) involved in the inflammatory response. Also, the benzyl group at the 4-position of the morpholine ring enhances lipophilicity, potentially improving membrane permeability and facilitating interaction with intracellular targets. The aromatic ring also offers the possibility of π-π stacking interactions with target proteins. Moreover, the butanoyl chain introduces flexibility and length between the morpholine core and the fluorophenoxy group. This spacer crucial for achieving the optimal distance and orientation for interaction with the binding site of the target protein. The length of the spacer influences the compound’s ability to reach and interact with specific regions of the target. The fluorophenoxy group introduces several important properties as Increases lipophilicity, potentially improving membrane permeability and bioavailability. So, the fluorine atom which is electron-withdrawing, which may subtly alter the electronic distribution within the molecule, potentially influencing its interaction with target proteins. This affect binding affinity and selectivity. Further, the fluorine substitution can enhance metabolic stability by reducing the susceptibility to enzymatic degradation. This led to a longer half-life and improved efficacy.

**Fig 1:**
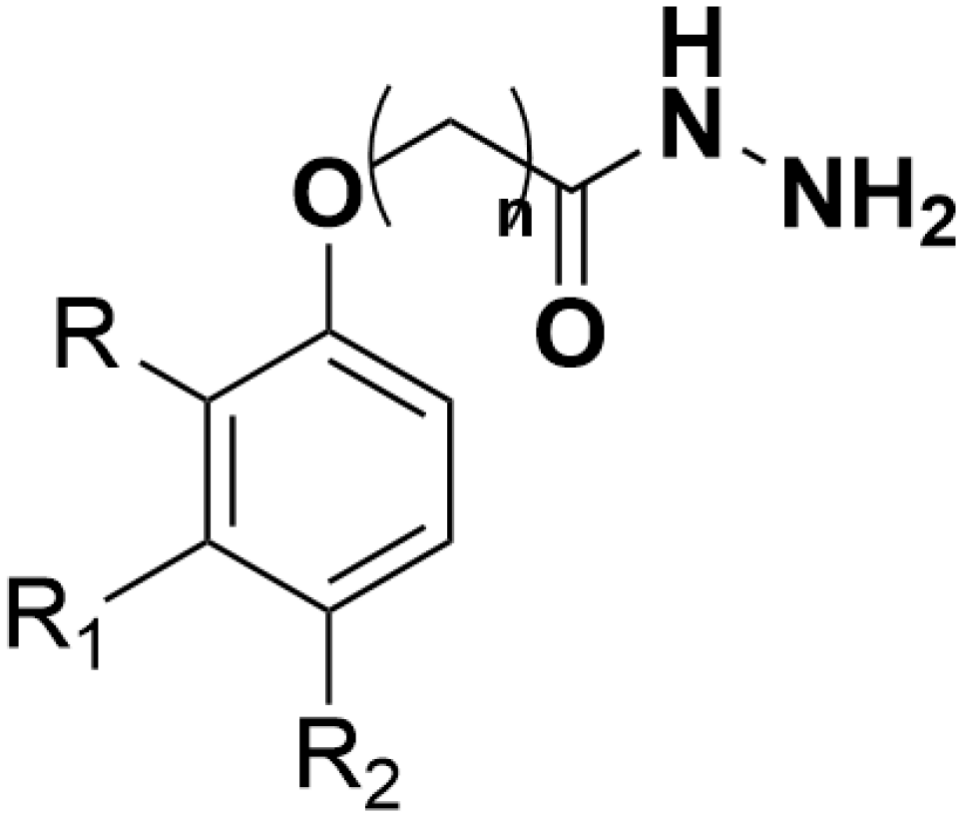
Chemical structure of 2-phenoxyacetohydrazide.

In contrast, these same substitutions were associated with a higher predicted probability of blood-brain barrier (BBB) penetration and P-glycoprotein inhibition, both of which may contribute to increased predicted toxicity and a higher likelihood of drug–drug interactions. Additionally, derivatives with multiple rotatable bonds and higher molecular flexibility tended to show reduced Caco-2 permeability and moderate acute toxicity predictions, as indicated in Table 1. These findings emphasize the need to balance binding affinity with pharmacokinetic and toxicity considerations during lead optimization. The compound **6e** might inhibit enzymes involved in the inflammatory cascade, such as cyclooxygenase (COX) or lipoxygenase (LOX) enzymes, thereby reducing the production of pro-inflammatory mediators like prostaglandins and leukotrienes. It modulates the activity of inflammatory receptors, such as chemokine receptors or cytokine receptors, thereby reducing the inflammatory response. In conclusion, the results indicate that the structure of 4-Benzyl-N’-(4-(4-fluorophenoxy)butanoyl)morpholine-2-carbohydrazide is very potent in modulating both disease processes, thus justifying their further investigation with preclinical models. In light of the complex interaction between inflammation and angiogenesis, this project endeavors to address the pressing need for multi-targeted therapies. For this purpose, structural optimization was carried out by introducing a morpholine group at the 3-position of the phenoxyacetic acid scaffold with the aim of enhancing the biological profile of the resulting hybrid compounds (Fig 1). These findings are an encouraging platform for further translational studies in chronic inflammatory diseases.

**Table 1:** Retrieved from the PDB database are the physiochemical characteristics of 1RV6.

Physiochemical Properties of Protein (1RV6)	
Parameters	Value
Cell Space Group	P 21 21 21
Crystallographic Resolution	2.5 Å
Molecular Weight	11.609 kDa
Amino-acid Chain Name	A, B (we used chain A)
Number of Amino-acid Residues	95

## Synthetic strategy and structural characterization of novel phenoxyacetohydrazide derivatives (6e-h)

**Scheme 1:**
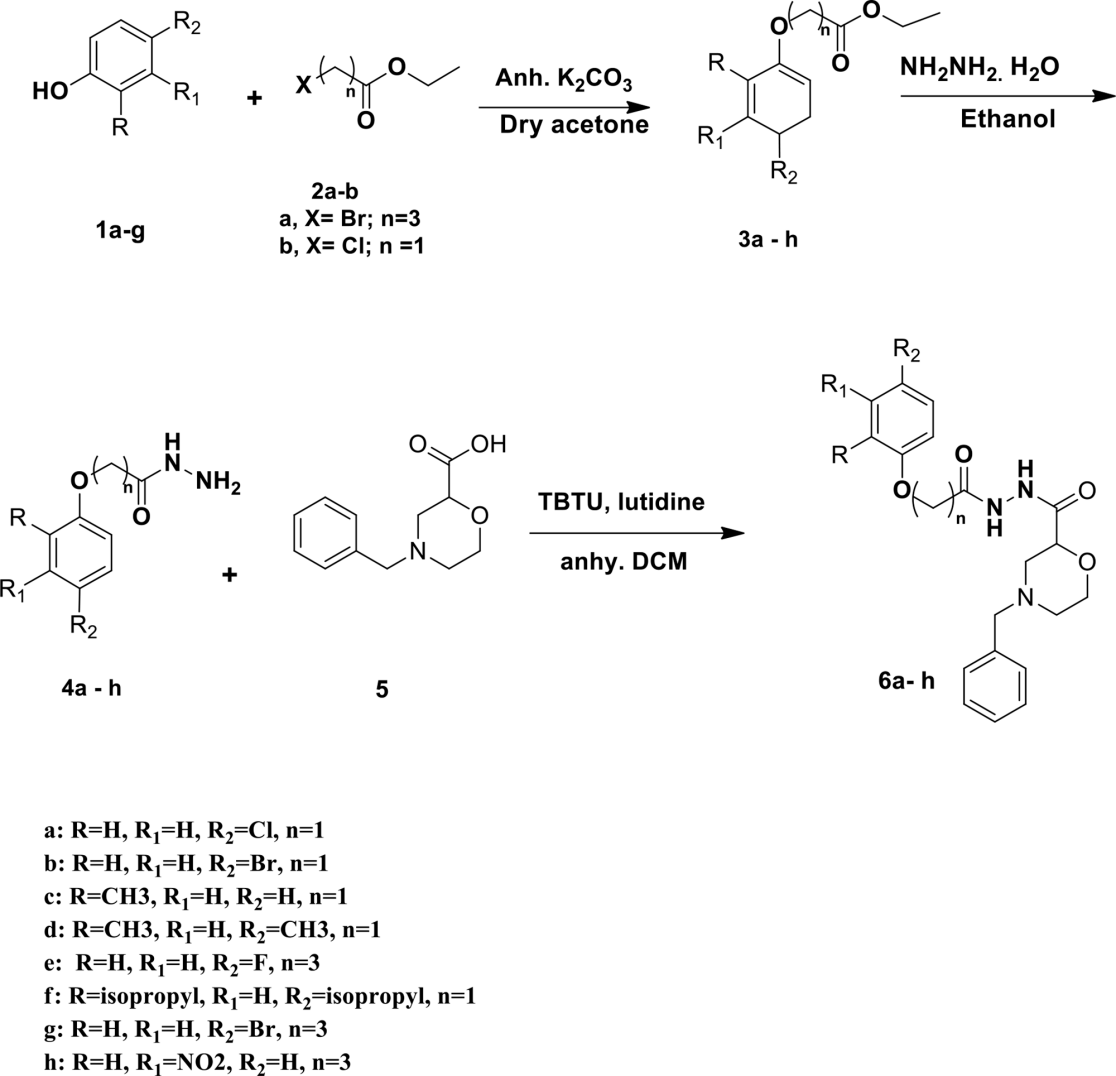
Synthesis of novel phenoxyacetohydrazide derivatives (6e-h).

The synthetic route to target phenyl 2-(4-benzylmorpholine-2-carbonyl) hydrazine-1-carboxylate **(6e-h)** were illustrated in Scheme 1. The first task was to prepare the key phenoxy acetic/ butyric acid ethyl esters **3a–h**. The key intermediates 3a–h were obtained by treatment of substituted phenols **1a–g** with substituted ester **2a–b**. The reaction of **3a–h** compounds with hydrazine hydrate in ethanol to form desired compounds 4a-h in good yields. Treatment of the hydrazide intermediates with 4-benzylmorpholine-2-carboxylic acid in presence of TBTU and lutidine as catalysts yielded phenyl 2-(4-benzylmorpholine-2-carbonyl) hydrazine-1-carboxylate (**6e-h**) in 72–94% yields.

## Investigating the impact of protein size and structure on the binding of compound 6e to VEGF, COX-1, and COX-2

This study investigated the potential interactions of compound **6e** with three target proteins: Vascular Endothelial Growth Factor (VEGF, PDB ID: 1RV6), Cyclooxygenase-1 (COX-1, PDB ID: 1EQG), and Cyclooxygenase-2 (COX-2, PDB ID: 1CVU). The docking studies were conducted using the Molecular Operating Environment (MOE) software package, with high-resolution X-ray crystal structures obtained from the Protein Data Bank (PDB).

Docking study outcomes are summarized qualitatively through visualizations and quantitatively through analysis of the physiochemical parameters enumerated in Tables 2 and 3. VEGF was greatly different in molecular weight (11.609 kDa) and number of residues (95 amino acids) compared to COX-1 (137.93 kDa, 580 residues) and COX-2 (132.6 kDa, 552 residues), showing differences in binding site complexity and ligand-binding specificity. Additionally, the crystal structures at high resolution (VEGF at 2.5 Å, COX-1 at 2.61 Å, and COX-2 at 2.40 Å) yielded dense structural backbones essential for accurate docking predictions, which are pivotal to the correct identification of interaction modes and binding site description.

**Table 2:** Retrieved from the PDB database are the physiochemical characteristics of 1EQG.

Physiochemical Properties of Protein (1EQG)	
Parameters	Value
Cell Space Group	P 21 21 21
Crystallographic Resolution	2.61 Å
Molecular Weight	137.93 kDa
Amino-acid Chain Name	A, B (we used chain A)
Number of Amino-acid Residues	580

**Table 3:** Retrieved from the PDB database are the physiochemical characteristics of 1CVU.

Physiochemical Properties of Protein (1CVU)	
Parameters	Value
Cell Space Group	P I 2 2 2
Crystallographic Resolution	2.40 Å
Molecular Weight	132.6 kDa
Amino-acid Chain Name	A, B (we used chain A)
Number of Amino-acid Residues	552

**Fig 2:**
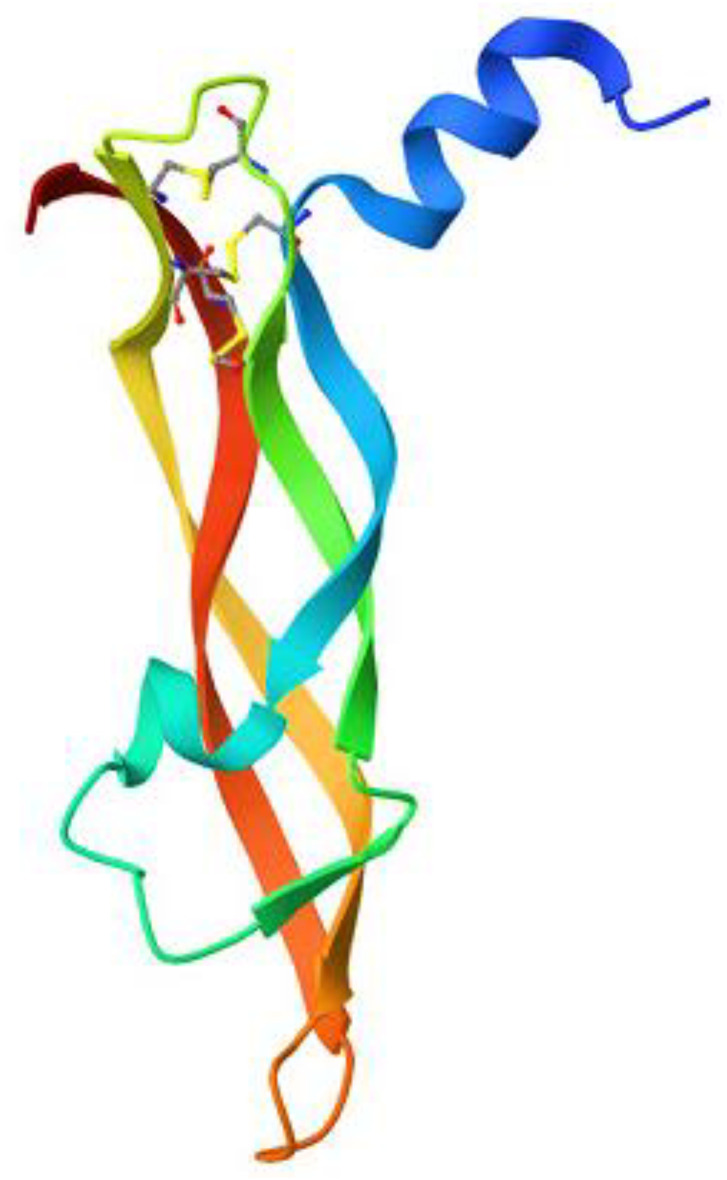
Ribbon representation of the crystal structure of vascular endothelial growth factor-A (VEGF-A), retrieved from the Protein Data Bank (PDB ID: 1RV6).

**Fig 3:**
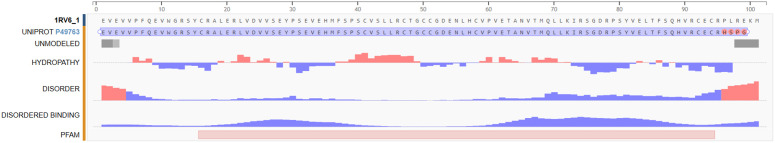
Visualization of the binding site of vascular endothelial growth factor-A (VEGF-A) (PDB ID: 1RV6).

**Fig 4:**
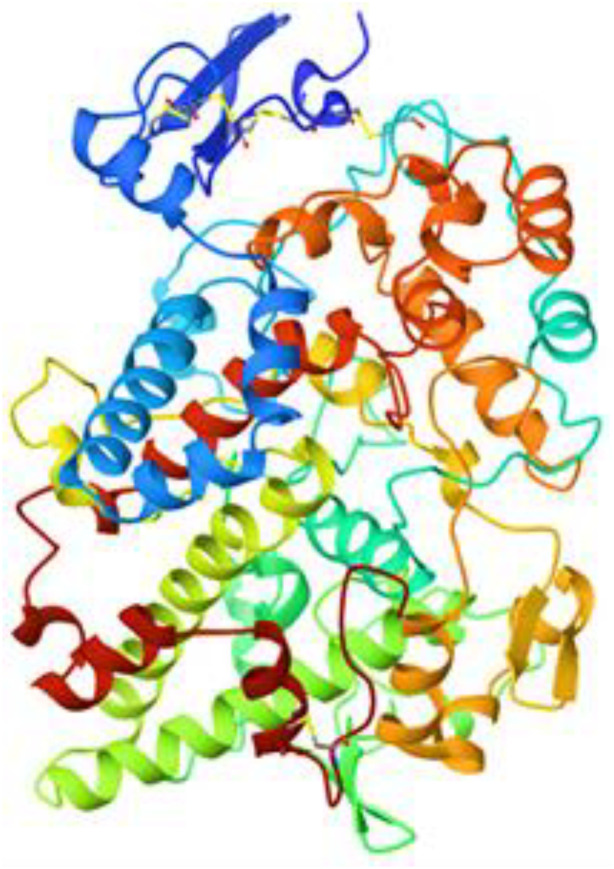
Ribbon representation of the crystal structure of cyclooxygenase-1 (COX-1) retrieved from the Protein Data Bank (PDB ID: 1EQG).

**Fig 5:**
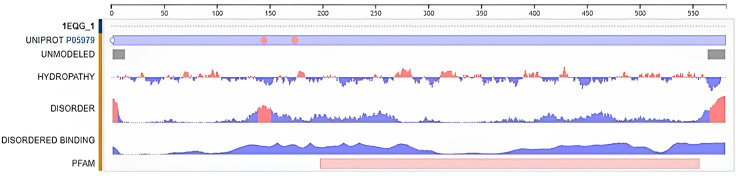
Visualization of the binding site of COX-1 (PDB ID: 1EQG).

**Fig 6:**
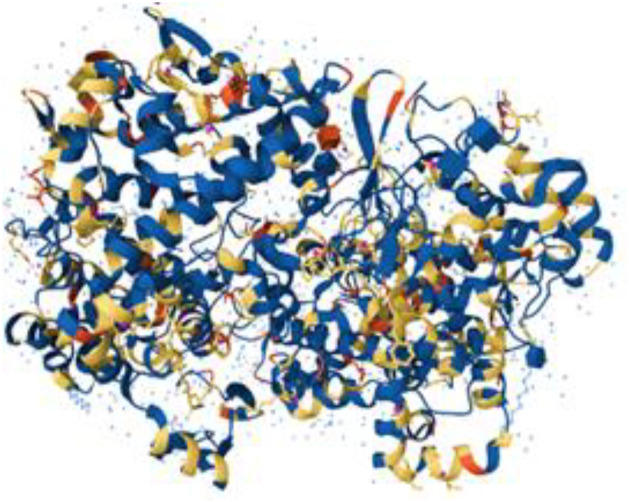
Ribbon representation of the crystal structure of cyclooxygenase-2 (COX-2) retrieved from the Protein Data Bank (PDB ID: 1CVU).

**Fig 7:**
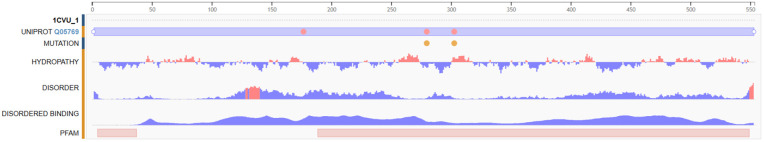
Visualization of the binding site of COX-2 (PDB ID: 1CVU).

Ribbon diagrams (Figs 2–4) showed the overall tertiary structures of target proteins, and close-up renderings of binding sites (Figs 5–7) emphasized the structural cavities where interactions with ligands are likely to take place. By visual inspection, it was possible to spot significant interaction differences and potential modes of binding. Specifically, the less complicated and lower binding cavity of VEGF predicted a likelihood of more specific and targeted ligand interactions with compound **6e**, whereas COX-1 and COX-2, with more complicated and larger structural topographies and alternative crystal packing schemes (space groups P 21 21 21 and P I 2 2 2, respectively), predicted several possibilities for binding and diverse interaction profiles. These in silico findings highlight the prospective therapeutic importance of compound **6e**. The important function of VEGF in angiogenesis and the involvement of COX enzymes in pain and inflammation processes render the selective binding profiles identified by bioinformatics-driven docking simulations to guide the following experimental validations towards prospective therapeutic applications to treat angiogenesis-related diseases and inflammatory and pain disorders.

## Evaluating the ADMET landscape of compound 6e: Balancing therapeutic potential and safety considerations

This study investigated the ADMET (Absorption, Distribution, Metabolism, Excretion, and Toxicity) along with properties under Lipinski’s Rule of Five (RO5) properties of compound **6e** (logP < 5, TPSA < 140 Å², Mwt > 500, hydrogen bond donors >5, and hydrogen bond acceptors >10 for oral bioavailability, and acceptable BBB penetration indicators) to assess its potential as a therapeutic agent. The results, summarized in Table 4, reveal a complex profile with both promising and concerning aspects. Compound **6e** (C₂₂H₂₆FN₃O₄) exhibits favorable physicochemical and ADMET properties supporting its potential as an orally bioavailable drug candidate. It has a moderate molecular weight (415.46 g/mol), acceptable LogP (2.75), and and acceptable topological polar surface area (TPSA, 79.90 Å²), suggesting good membrane permeability. The calculated LogS values (−3.45 and −6.42, from different prediction models) indicate moderate to low aqueous solubility, although both Silicos-IT and Ali class predictions classify it as soluble. this limitations of in silico predictions need for experimental validation.

**Table 4:** Comprehensive ADMET profile of compound 6e.

Properties	Molecule
	6e
**Formula**	C_22_H_26_FN_3_O_4_
**MW**	415.46 g/mol
**Heavy atoms**	30
**Aromatic heavy atoms**	12
**Rotatable bonds**	11
**H-bond acceptors**	6
**H-bond donors**	2
**Fraction Csp3**	0.36
**MR**	112.43
**TPSA**	79.90 Å²
**Consensus Log P**	2.53
**Octanol/water partition coefficient(logP)**	2.75
**Aqueous solubility LogS**	−3.45
**Ali Class**	Soluble
**Silicos-IT LogS**	−6.42
**Silicos-IT class**	Soluble
**log Kp (cm/s)**	−7.18
**Lipinski #violations**	0
**Bioavailability Score**	0.55
**GPCR ligand**	−3.65
**Molecular refractivity**	112.43

With zero Lipinski violations and a bioavailability score of 0.55, it demonstrates suitable drug-likeness. Its molecular refractivity (112.43) and 11 rotatable bonds indicate potential for favorable target interactions. ADMET predictions indicate high human intestinal absorption (HIA + , 0.9475) and blood-brain barrier penetration (BBB + , 0.9113), though the low Caco-2 permeability (0.7022) introduces uncertainty. However, the prediction suggests low Caco-2 permeability (Caco2-, 0.7022 probability), which seems contradictory to the HIA prediction and warrants further investigation. The carcinogenicity prediction is non-required (0.5288 probability), indicating a moderate uncertainty in this aspect. The acute oral toxicity prediction classifies it as class III (0.6673 probability), suggesting moderate toxicity. The predicted LD50 (2.4760 mol/kg in rats) requires careful consideration and experimental validation as shown in Table 5.

**Table 5:** Toxicity profile analysis of compound 6e insights from ADMET “SAR” predictions for safer drug development.

Model	6e	
**Blood-Brain Barrier**	Result	BBB+
	Probability	0.9113
**P-glycoprotein Inhibitor**	Result	Inhibitor
	Probability	0.8986
**Human Intestinal Absorption**	Result	HIA+
	Probability	0.9475
**Caco-2 Permeability**	Result	Caco2-
	Probability	0.7022
**Carcinogenicity (Three-class)**	Result	Non-required
	Probability	0.5288
**Acute oral toxicity**	Result	III
	Probability	0.6673
**Rat Toxicity LD50 mol/kg**	Result	LD50, mol/kg
	Probability	2.4760

### Evaluation the network connections, toxicity profile, and distribution characteristics of the compound 6e.

**Fig 8:**
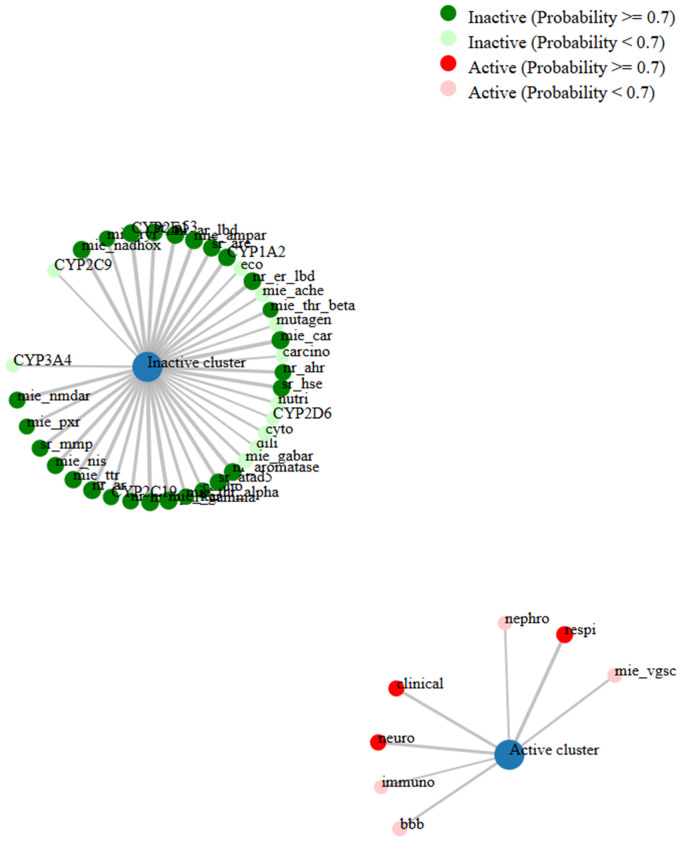
Network diagram illustrating the predicted pharmacological activity profile of the 6e compound.

**Fig 9:**
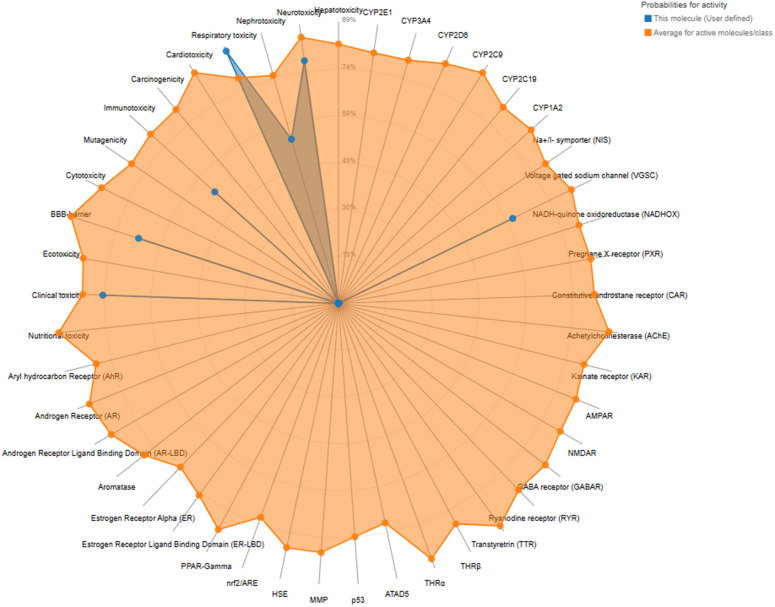
Toxicity radar chart representing the predicted toxicity profile of the 6e compound.

**Fig 10:**
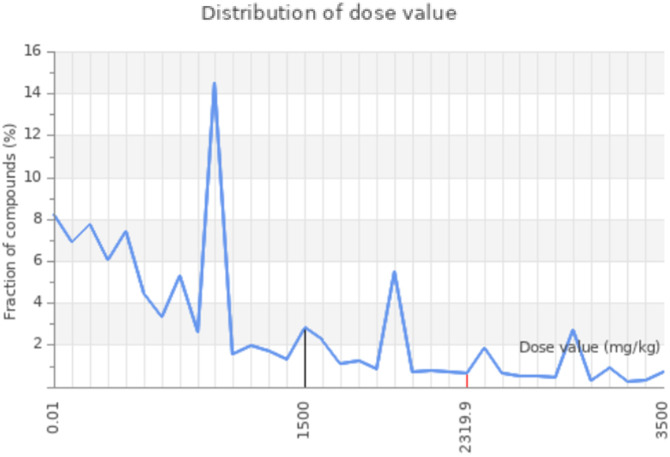
Distribution of dose value of the 6e compound.

**Fig 11:**
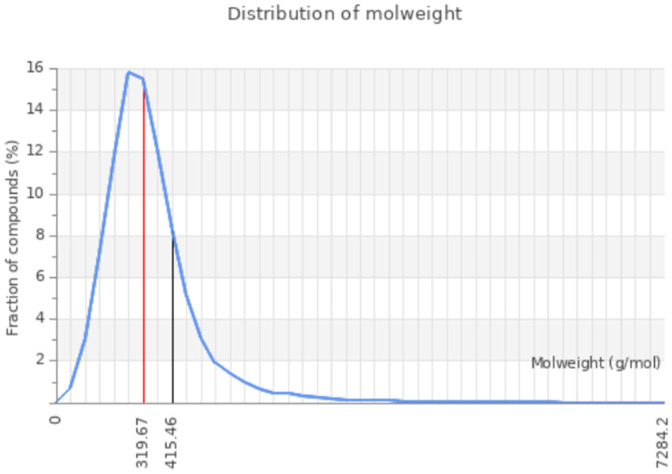
Distribution of molecular weight value of the 6e compound.

The network chart (Fig 8) illustrates the connection between the compound **6e** and its predicted activities. This chart suggests that compound **6e** is linked to multiple activities, indicating its potential as a multifunctional agent. The nodes in the chart show the predicted activities, while the edges show the relations between the compound and the activities. The chart gives a graphical idea of the possible applications and interactions of the compound. Moreover, the toxicity radar chart indicates the level of confidence in positive toxicity results for compound **6e** with respect to the average of its classification. Fig 9 Compound **6e**, which displays a relatively low toxicity profile when compared to the class average, is shown here. The radar chart is a very effective and simple way to visualize the toxicity profile of the compound, allowing for the quick identification of potential problems. The dose value range for compound **6e** also defines the range of doses over which the compound exhibits its activity. The chart shows that compound **6e** elicits activity across a range of dosages and shows peak activity at one of the dosages. The dose value analysis provides important information useful in extrapolating compound **6e**’s application into various fields, as exemplified by Fig 10. The molecular weight distribution of compound **6e** also elucidates the spectrum of molecular weights corresponding to its biological activity. The data illustrated in this graph show that compound **6e** exhibits activity over the whole range of molecular weights and attains its complete effectiveness at a certain molecular weight. Just by the spread in values of the molecular weight, one realizes enormous potential toward the optimization of various applications of compound **6e**, as portrayed in Fig 11. More importantly, all the results raise significant understanding toward the characteristics and expected activities related to compound **6e** itself. Taken together, the network diagram, toxicity radar plot, and dispersion of dosage and molecular weight metrics give a better understanding of the possible applications and constraints of the compound. Such findings can direct further studies and applications of compound **6e**, hence setting a ground for further research and development.

## Construction and preparation of the compound 6e

**Fig 12:**
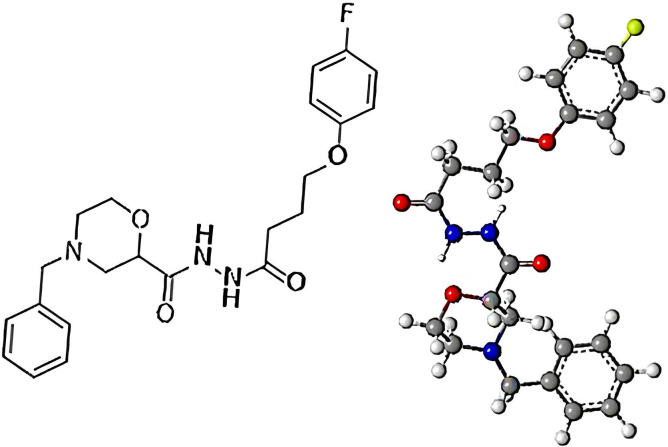
Chemical structural of the compound 6e. The initial 2D structure was optimized in MOE and converted into a 3D model using Chem3D 16.0.

This study was focused on designing and preparing compound **6e** in order to increase the binding affinity and explore their therapeutic potential. The initial 2D structures of the compound **6e** was created using ACD/ChemSketch, providing a blueprint that would be essential in the next steps. This phase emphasized the incorporation of specific structural features that would increase binding and pharmacological activity. Then, the 2D structure was imported into MOE for preparation. Key parameters included ensuring correct ionization states of oxygen atoms at physiological pH, which is important to accurately represent the behavior of the compound **6e** in biological contexts. Optimization also involved adding and removing hydrogen atoms in an attempt to further enhance binding interactions. Finally, the last step was to convert the 2D structure of compound **6e** to 3D representations using Chem3D 16.0. This conversion is very important in visualizing the ligand orientation and possible interactions in the active site of the target protein. The final 3D structure of compound **6e** is shown in Fig 12.

### Compound 6e exhibits multifaceted binding interactions with VEGF, COX-1, and COX-2 as revealed through molecular docking analysis.

The current study attempts an extensive *in silico* molecular docking evaluation of compound **6e** against the three principal receptors: VEGF (PDB ID: 1RV6), COX-1 (PDB ID: 1EQG), and COX-2 (PDB ID: 1CVU). To see the general binding trends, we docked compound 6e into the target proteins and compared them with the standard NSAID drug indomethacin. The docking results of compound **6e** within the active site of VEGF receptor formed two hydrogen bonds with Pro1041 and Lys861. On the other hand, the docking results of compound within the active site of cyclooxygenase-1 (COX-1) formed one hydrogen bonds with His4. Finally, the docking results of compound 6e within the active site of cyclooxygenase-2 (COX-2) formed one hydrogen bonds with Tyr 130. The docking studies reveal some outstanding binding affinities, therefore suggesting that compound **6e** could act as an inhibitor of the selected targets. The docking assessment resulted in negative score values for each of the three receptors, suggesting substantial binding affinities. In particular, the VEGF receptor demonstrated a docking score of **−13.1622** (kcal/mol), indicative of a strong interaction with compound **6e**. as opposed to the binding energy with indomethacin **−9.2719** (kcal/mol).

**Fig 13:**
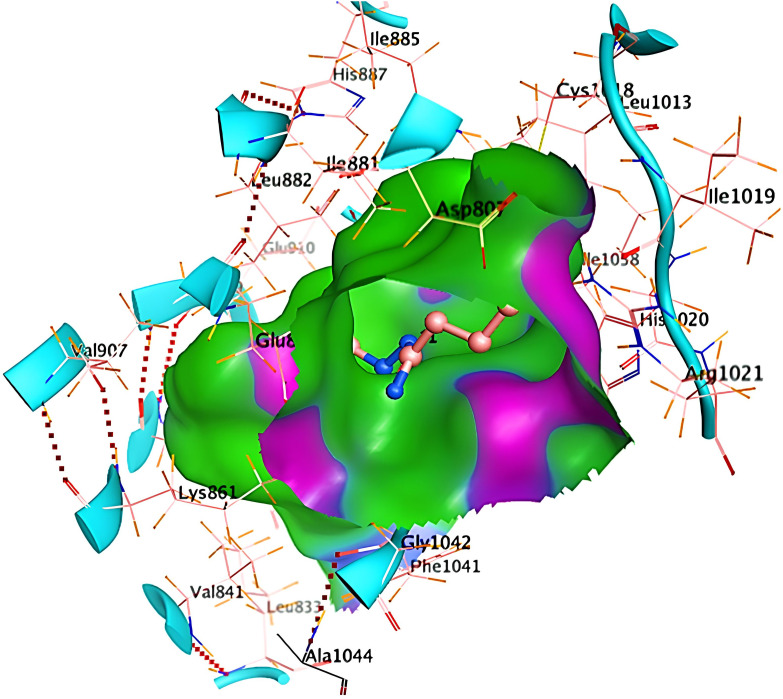
A detailed 3D visualization demonstrating the binding interaction of compound 6e (depicted in blue and red) within the active site of the VEGF receptor (PDB ID: 1RV6), represented by green ribbon structures. This illustration highlights the precise fit of compound **6e** into the binding pocket, akin to a key fitting into a lock, emphasizing its potential as a therapeutic agent.

**Fig 14:**
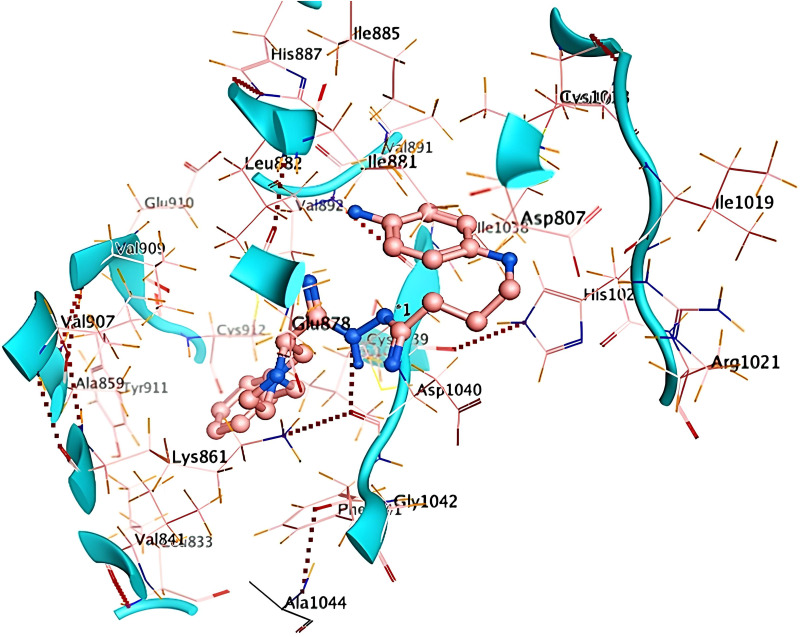
A detailed 2D schematic representation illustrating the non-covalent interactions that stabilize the VEGF receptor (PDB ID: 1RV6)-compound 6e complex. This visualization highlights the key hydrogen bonds, hydrophobic interactions, and other stabilizing forces contributing to the assembly’s structural integrity and binding affinity.

**Fig 15:**
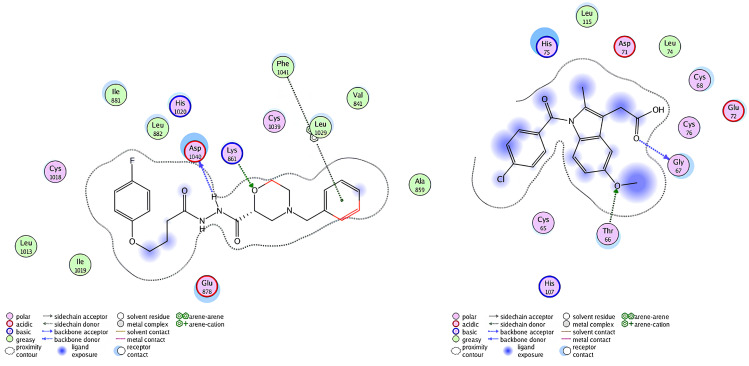
2D interaction of compound 6e (left) and indomethacin drug (right) within the active site of VEGF receptor (PDB ID: 1RV6).

**Fig 16:**
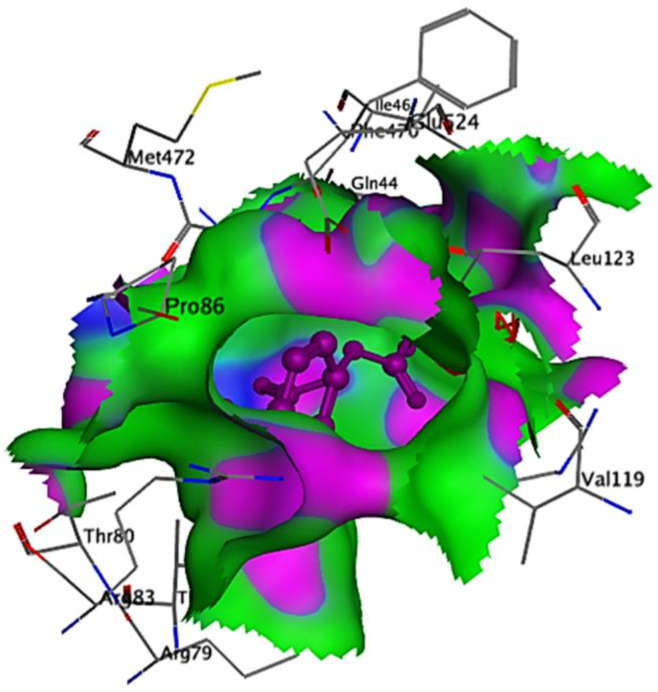
A captivating 3D visualization showcasing the binding interaction of compound 6e (depicted in red) within the active site of the COX-1 receptor (PDB ID: 1EQG).

**Fig 17:**
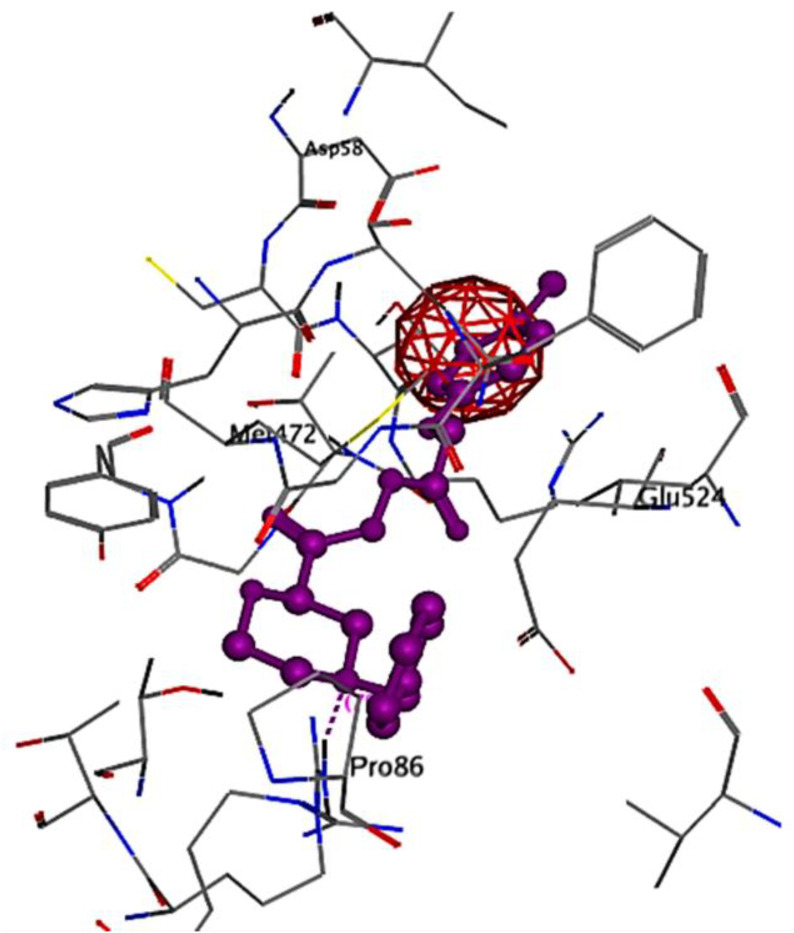
A detailed 2D schematic representation illustrating the non-covalent interactions that stabilize the COX-1 receptor-6e complex.

**Fig 18:**
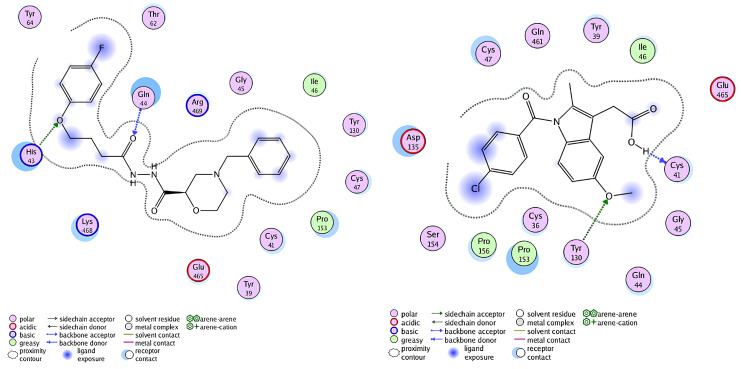
2D interaction of compound 6e (left) and indomethacin drug (right) within the active site of cyclooxygenase-1 (COX-1) (PDB ID: 1EQG).

**Fig 19:**
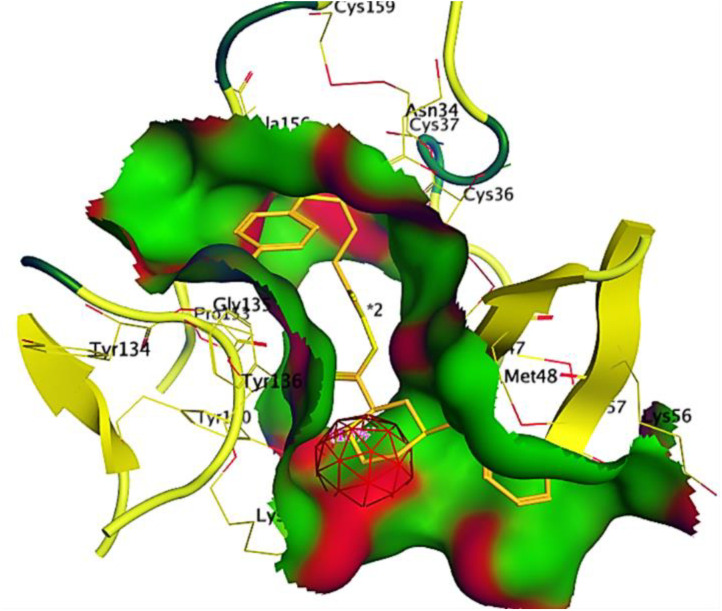
A striking 3D visualization showcasing the binding interaction of compound 6e (depicted in red) within the active site of the COX-2 receptor (PDB ID: 1CVU).

**Fig 20:**
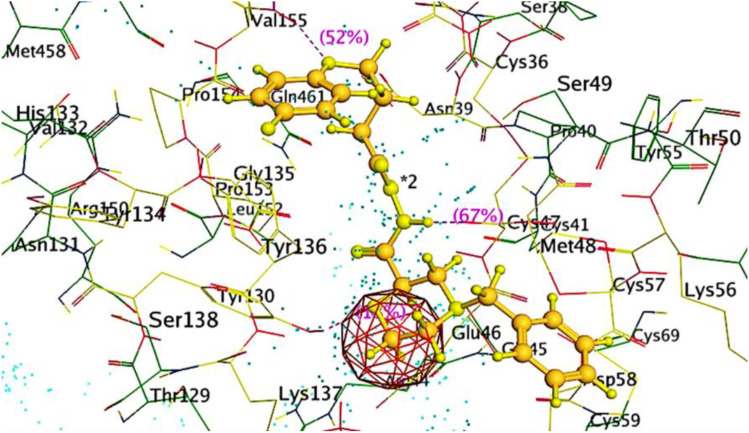
A detailed 2D schematic representation illustrating the non-covalent interactions stabilizing the COX-2 receptor (PDB ID: 1CVU)-compound 6e complex.

**Fig 21:**
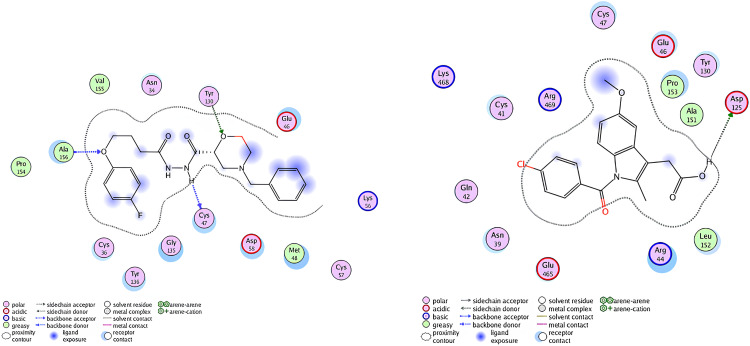
2D interaction of compound 6e (left) and indomethacin drug (right) within the active site of cyclooxygenase-2 (COX-2) (PDB ID: 1CVU).

This score signifies advantageous binding circumstances, implying that compound **6e** is proficient in inhibiting VEGF, an essential regulator of angiogenesis. This resultant strong binding suggests therapeutic applications in diseases where VEGF plays a pivotal role, such as cancer. In contrast, COX-1 and COX-2 displayed docking scores of **−12.5301** and **−12.6705** (kcal/mol), respectively. These scores also indicate high binding affinities compared to those of indomethacin which been **−11.7255** and **−12.5679** (kcal/mol) respectively. The binding interactions with COX-1 and COX-2 suggest that compound **6e** may influence inflammatory processes, given the roles of these cyclooxygenases in pain and inflammation pathways. The similar docking scores for COX-1 and COX-2 indicate that compound **6e** exhibit comparable inhibitory effects on both enzymes, although the specific biochemical consequences would require further experimental validation. Figs 13–15 provide visual insights into the docking interactions of compound **6e** with the VEGF receptor. The 3D representation in Fig 13 illustrates how compound **6e** fits into the VEGF binding pocket, akin to a key fitting into a lock. This visualization underscores the potential for compound **6e** to effectively occupy the binding site, thereby inhibiting VEGF activity. Fig 14 complements this by showcasing a 2D schematic that elucidates the non-covalent interactions stabilizing the VEGF-**6e** complex. These interactions are critical for maintaining the binding affinity and ensuring the efficacy of compound **6e** as a potential inhibitor. The ball-and-stick model in Fig 15 provides a detailed depiction of the molecular engagement, highlighting the specific atoms involved in the binding process. Similarly, Figs 16–18 illustrate the interactions between compound **6e** and COX-1. The visualizations confirm a compatible fit within the COX-1 binding pocket, indicating that compound **6e** can effectively interact with the enzyme. The non-covalent bonding interactions are again depicted in the 2D schematic (Fig 17), which is essential for the stabilization of the receptor-ligand complex. For COX-2, Figs 19–21 present similar representations. The fit of compound **6e** within the COX-2 binding pocket is visually compelling, demonstrating that **6e** occupies a critical space for effective binding. These figures collectively emphasize the potential of compound **6e** as a dual inhibitor of both COX-1 and COX-2, which could have significant implications for therapeutic strategies targeting inflammation. The docking results presented in Tables (1S, 2S, and 3S in S1 File) further elaborate on the binding energies and inhibition constants of compound **6e** with the considered receptors. Notably, compound **6e** demonstrated very low binding energy during complex formation with VEGF, along with a relatively low inhibition constant value, suggesting its capability as a VEGF inhibitor. Moreover, data demonstrate that compound **6e** is capable of binding to cyclooxygenases, with stable interactions in both COX-1 and COX-2. These results of the docking study provide evidence to indicate that compound **6e** can be considered a potential candidate for further evaluation in terms of both VEGF and cyclooxygenase inhibition. The strong binding affinities infer that the compound **6e** may strikingly affect the functionality of these receptors, which are involved in critical biological processes such as angiogenesis and inflammation. Given that VEGF is an essential factor in tumor-related angiogenesis, the ability of compound **6e** to block this receptor could make it a potential candidate for therapeutic intervention in the area of cancer therapy. Moreover, the inhibition of COX-1 and COX-2 has also been well documented in the treatment of pain management and inflammatory disorders, respectively, so compound **6e** could likewise be expected to exhibit analgesic activity.

### *In Vitro* anti-inflammatory activity: Human red blood cell (HRBC) membrane stabilization assay.

An in vitro HRBC membrane stabilization assay was conducted to evaluate the anti-inflammatory potential of eight synthesized compounds (6a-h). The assay measures the ability of a compound to prevent the lysis of HRBC membranes under stress, indirectly indicating anti-inflammatory activity. Results are expressed as “IC50” values (µg/ml), representing the concentration needed to inhibit 50% of membrane lysis. Lower “IC50” values indicate greater potency. Compound **6e** demonstrated the most potent membrane stabilization with an “IC50” of **155** µg/ml. Compounds 6b (222 µg/ml) and 6d (199 µg/ml) also showed significant activity. The remaining compounds (6a, 6c, 6f, 6g, 6h) exhibited weaker activity (“IC50” values ranging from 255 µg/ml to 311 µg/ml). Further studies, including in vivo assays and mechanistic investigations, are needed to confirm these findings and elucidate the compounds’ anti-inflammatory mechanisms. Structure-activity relationship “SAR” analysis of compounds 6a-h may reveal structural features contributing to their potency*.* (Table 6)

**Table 6:** IC_50_ values (μg/mL) of compounds 6e–6h determined using the human red blood cell (HRBC) membrane stabilization assay (One-way ANOVA, *p* < 0.0001).

Samples	HRBC membrane stabilization “IC50” Value (µg/ml)
Control	–
6a	311
6b	222
6c	289
6d	199
6e	155.199
6f	301
6g	279
6h	255

Moreover, the anti-inflammatory potential of compound **6e** was evaluated using the HRBC membrane stabilization assay. This well-established in vitro method assesses the ability of a compound **6e** to inhibit the lysis of HRBC membranes under stressed conditions, which serves as an indirect measure of anti-inflammatory activity. The results of the HRBC membrane stabilization assay for compound **6e** are presented in Table 7. The assay was conducted using different concentrations of **6e** (50, 100, 150, 200, and 250 μg) and the standard anti-inflammatory drug indomethacin for comparison. Compound **6e** showed a concentration-dependent increase in membrane stabilization, with percent inhibition ranging from 26.68% at 50 μg to 68.65% at 250 μg. **Also, t**he calculated “IC50” value for compound **6e** was 164.296 μg/ml. This indicates the concentration required to inhibit 50% of HRBC membrane lysis. While, the standard anti-inflammatory drug indomethacin exhibited a lower “IC50” value of 121.68 μg/ml, suggesting it has a higher potency in stabilizing HRBC membranes compared to compound **6e**.

**Table 7:** *in-vitro* anti-inflammatory activity of compound 6e. Data are shown as mean ± (n = 6). (Tukey’s multiple range, post hoc test, p < 0.148).

Sample	Concentration (µg)	Absorbance at 560nm	% Inhibition	“IC50” (µg/ml)
Control	–	0.912 ± 0.012	–	
Indomethacin	50 100 150 200 250	0.601 ± 0.003 0.501 ± 0.001 0.398 ± 0.001 0.298 ± 0.002 0.188 ± 0.008	33.10 46.11 53.99 64.98 76.94	122.01
Compound 6e	50 100 150 200 250	0.702 ± 0.001 0.634 ± 0.002 0.601 ± 0.007 0.449 ± 0.004 0.389 ± 0.002	28.88 34.13 42.02 59.99 67.86	165.988

The results from the HRBC membrane stabilization assay demonstrate that compound **6e** possesses significant anti-inflammatory activity. The concentration-dependent increase in membrane stabilization and the relatively low “IC50” value indicate that **6e** can effectively inhibit the lysis of HRBC membranes under stress conditions. The HRBC membrane stabilization assay provides a useful in vitro model to assess the anti-inflammatory potential of **6e**,

### Compound 6e exhibits potent anti-angiogenic effects by inhibiting VEGF-Induced vascular formation.

**Fig 22:**
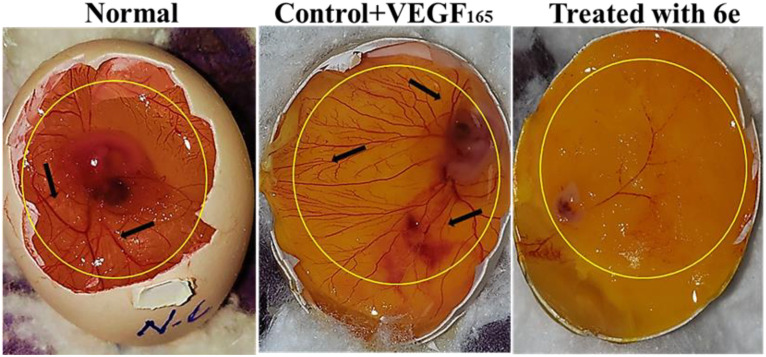
Effect of 6e on rVEG F165 induced in vivo CAM. images of the chorioallantoic membrane (CAM) from fertilized chicken eggs after treatment. Left image (Negative Control): Dense and highly branched neovasculature (black arrows) visible within the yellow demarcation zone, indicating active angiogenesis. Middle image (Positive Control or Standard Treatment): Moderate reduction in vessel density and branching. Right image (Compound 6e-treated group): Marked suppression of neovascularization with sparse, thin, and poorly branched blood vessels, demonstrating significant anti-angiogenic potential.

**Fig 23:**
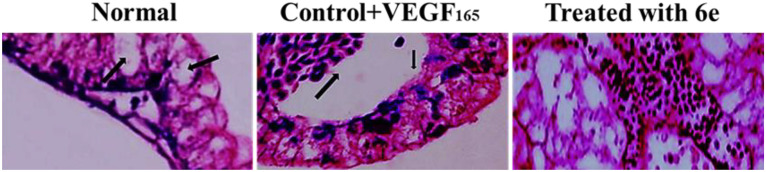
H&E staining of *in vivo* CAM. Representative histological sections of corneal tissue from experimental groups stained with hematoxylin and eosin: Left image (Negative control): Extensive neovascularization (black arrows) and inflammatory cell infiltration are observed in the stromal layer. Middle image (Indomethacin-treated group): Moderate reduction in neovascular structures (black arrows) and inflammatory cells, with partially restored corneal architecture. Right image (Compound 6e-treated group): Marked improvement in corneal structure with minimal inflammatory infiltration and absence of neovessel, indicating significant anti-inflammatory and anti-angiogenic effects.

**Fig 24:**
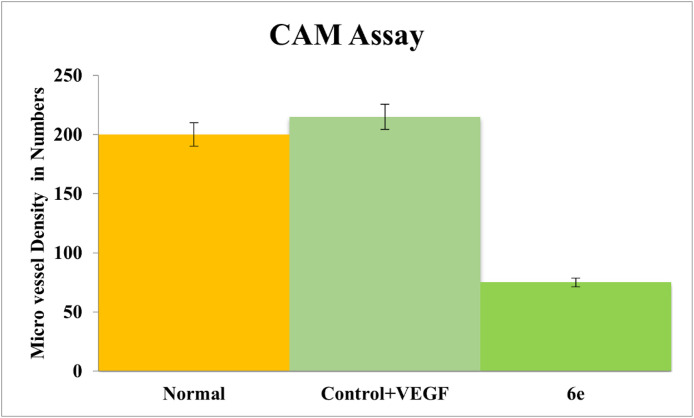
Total vessel length of *in vivo* CAM. Each value is expressed as mean ± SD (*p* < 0.001 versus control, n = 6).

**Fig 25:**
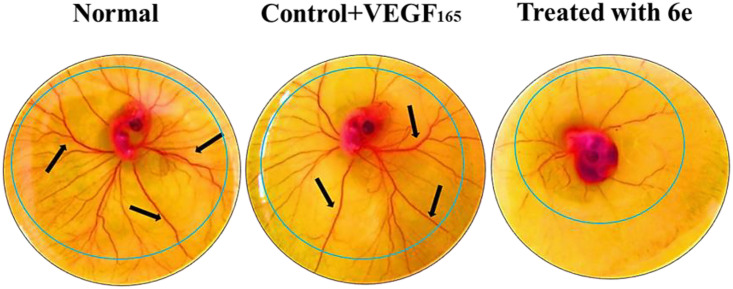
Effect of 6e on rVEG F165 induced ex-in vivo CAM assay. Left image (Control + VEGF): Dense and highly branched vasculature (black arrows) radiating from the central embryo, indicating robust VEGF-induced angiogenesis. Middle image (Standard treatment): Moderate reduction in vessel branching and density, consistent with partial anti-angiogenic activity. Right image (Compound 6e-treated group): Marked suppression of new blood vessel formation with sparse and thin vascular branches, indicating significant anti-angiogenic potential of compound **6e**.

**Fig 26:**
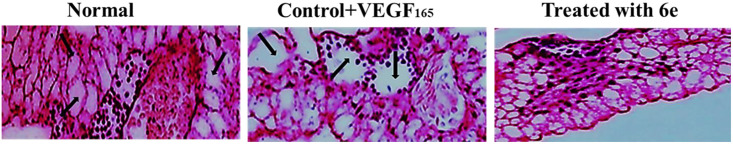
H&E staining of *ex-vivo* CAM. Left image (Control + VEGF): Extensive neovessel formation (black arrows) and infiltration of inflammatory cells into the mesodermal layer, indicating strong pro-angiogenic response. Middle image (Standard treatment): Moderate vascular proliferation and inflammation with partial normalization of tissue architecture. Right image (Compound **6e**-treated group): Significant reduction in blood vessel formation and absence of inflammatory infiltrate, suggesting strong anti-angiogenic and anti-inflammatory effects of compound **6e**.

**Fig 27:**
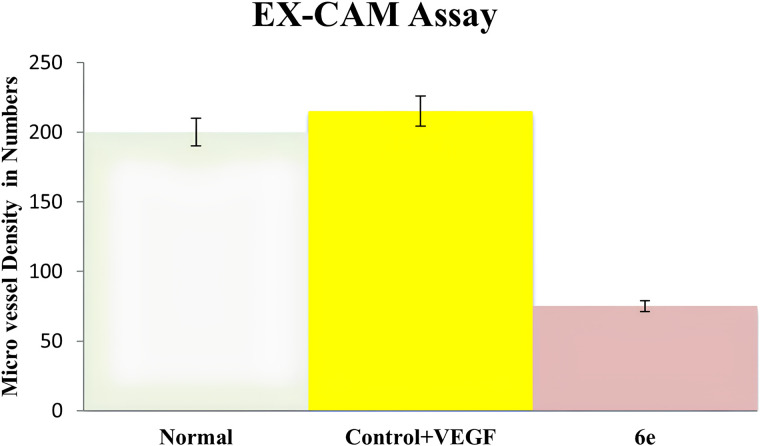
Total vessel length of *ex-vivo* CAM. Each value is expressed as mean ± SD (p < p < 0.0001versus control, n = 6).

The effects of compound **6e** on VEGF165-stimulated angiogenesis were examined both *in vivo* and *ex vivo* by using the chick chorioallantoic membrane (CAM) assays. As depicted in Figs 22–27, these results suggests that Compound **6e** demonstrates anti-angiogenic activities through its impact on vascular development and some of the crucial molecular pathways involved. The CAM assay is a well-developed model for the evaluation of pro- and anti-angiogenic activities of compounds. The results indicate that treatment with recombinant VEGF165 (rVEGF165) significantly increased vascularization of the CAM compared to the untreated control. However, co-administration of compound **6e** inhibited this rVEGF165-induced angiogenic response in a dose-dependent manner. This inhibitory effect aligns with the role of VEGF in pathological angiogenesis, signifying that **6e** may act as a promising VEGF pathway inhibitor, with potential applications in treating diseases like inflammation where VEGF-driven angiogenesis is a hallmark (Fig 22).

Fig 23 presents the Hematoxylin and Eosin (H&E) staining of the *in vivo* CAM samples. The staining was performed to visualize and quantify the blood vessel formation in response to the treatment. The control CAM showed a basal level of blood vessel density, as indicated by the positive staining for endothelial cell markers. This finding is consistent with the anti-angiogenic activity of **6e** observed suggests that **6e** can effectively inhibit VEGF-induced angiogenesis. Furthermore, quantitative analysis in Fig 24 confirms the inhibitory effect of 6e on total vessel length, a key metric of angiogenesis. A significant reduction in vessel length was observed in treated CAMs compared to control, supporting the qualitative findings from Fig 22. Interestingly, the reduction appeared to be dose-dependent, with higher concentration of **6e** leading to more pronounced inhibition. This suggests that **6e** not only inhibits the initiation of new blood vessels but also impacts the elongation and maturation of existing vessels. Such dual effects on angiogenesis strengthen its therapeutic potential. Moreover, these findings are consistent with the proposed mechanism of VEGF pathway inhibition, as VEGF is crucial for both early and late stages of angiogenesis.

Also, Fig 25 depicts the results of the *ex vivo* CAM assay, which allows for the evaluation of angiogenic responses in a more controlled and isolated ex vivo environment.

Similar to the *in vivo* results, the *ex vivo* CAM assay showed that rVEGF165 treatment significantly enhanced the vascularization of the CAM explants. In contrast, the co-administration of compound 6e effectively inhibited this VEGF-induced angiogenic response in a dose-dependent manner. More, the H&E staining of the ex vivo CAM samples, shown in Fig 26, corroborates the findings from the in vivo CAM assay. In addition, the quantitative analysis of the total vessel length in the ex vivo CAM assay, presented in Fig 27, further supports the anti-angiogenic activity of compound 6e. The rVEGF165-induced increase in total vessel length was significantly attenuated by the addition of **6e** in a dose-dependent manner.

In conclusion, the results from the *in vivo* and *in vivo* CAM assays, together with supporting quantitative and hormonal analyses, demonstrate that compound 6e possesses potent antiangiogenic properties. Compound 6e was able to effectively inhibit VEGF-induced angiogenesis, as demonstrated by a reduction in vessel density and total vessel length in both *in vivo* and *ex vivo* models. These data suggest that compound **6e** may have therapeutic potential in disease states characterized by pathological angiogenesis, such as cancer and some inflammatory disorders.

## Anti-inflammatory and antiangiogenic effects of compound 6e in a CNV of rat model

**Fig 28:**
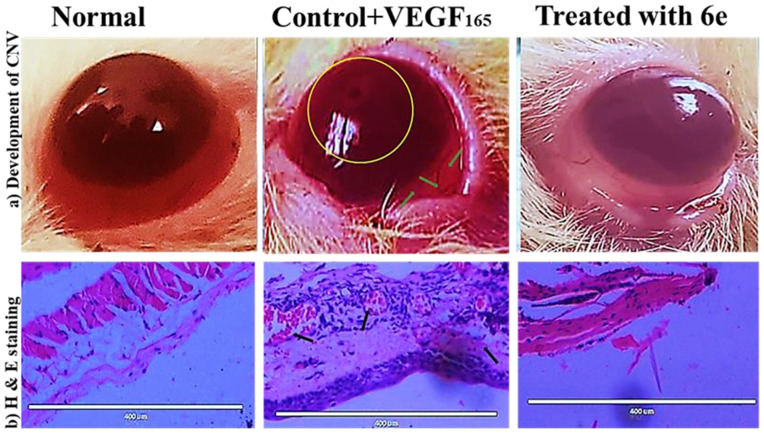
Angiogenesis modulatory effect of compound 6e on neovascularization (a) Representative photographs of the rat cornea illustrating the inhibition of angiogenesis in alkali-burnt corneas following treatment with compound 6e. (b) Hematoxylin and eosin (H&E) staining of the cornea reveals improved corneal structure and morphology after treatment with compound **6e**. (c) Quantitative analysis showing a reduction in total vessel length in the rat cornea after treatment with compound **6e**, demonstrating its efficacy in modulating angiogenesis.

The corneal alkali burn model is a well-established and highly reliable experimental tool for the assessment of pharmacological agents’ effects on inflammatory angiogenesis. As early as three days after the corneal alkali injury, the development of CNV was seen in both the control and the compound **6e**-treated group, as shown in Fig 28. However, the corneas in the compound **6e** treatment group showed a significant decrease in CNV compared with the control corneas. The results presented in the present study provide strong proof that the compound **6e** shows high angiogenesis-modulating activities, which resulted in a reduced neovascularization in the rat cornea model. Inhibition of angiogenesis, as illustrated by the representative images in Fig 28a and 28b, indicated that compound **6e** was able to effectively reduce the formation of new blood vessels in the alkali-burnt cornea. Results are further supported by H&E staining, which shows an improved corneal morphology, suggesting that the compound has a positive effect on corneal integrity and health. Moreover, the quantitative analysis shown in Fig 28c supports the antiangiogenic properties of compound **6e**. The reported diminution in the total vessel length after treatment speaks to a significant reduction in neovascularization, an integral trait of the potential of compound **6e** on the angiogenic process. These results have important implications for the potential therapeutic applications of compound **6e** in the management of CNV, a common complication associated with various ocular conditions, such as corneal injuries, infections, or inflammatory disorders. By effectively inhibiting the formation of new blood vessels, compound **6e** contribute to the preservation of corneal clarity and visual function, ultimately enhancing the treatment options for patients suffering from these conditions. Nonetheless, the findings presented in this study provide a strong foundation for the continued development and exploration of compound **6e** as a promising therapeutic candidate for the management of CNV.

## Anti-edema effect of compound 6e on carrageenan-induced paw edema in rats

**Fig 29:**
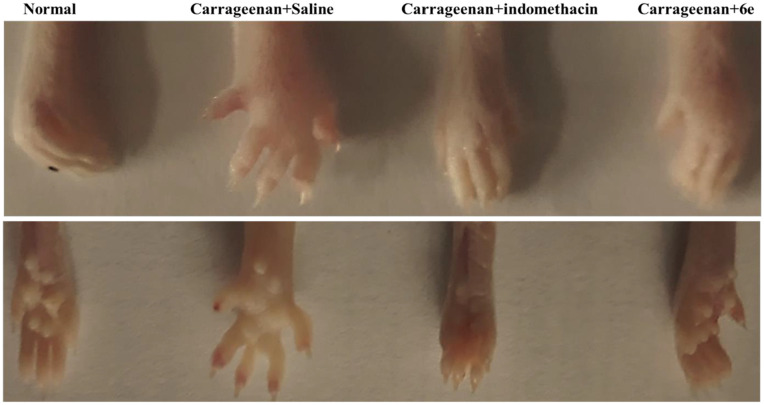
Photograph of representative rat of each group showing the effect of treatment on paw edema treated with compound 6e.

**Fig 30:**
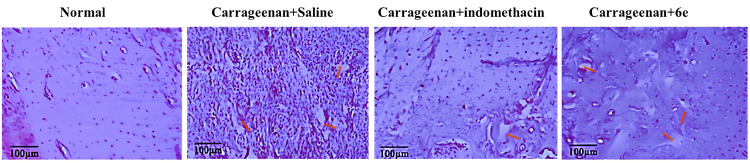
H and E staining representative rat changes of each group showing the effect of treatment on paw edema treated with compound 6e.

**Fig 31:**
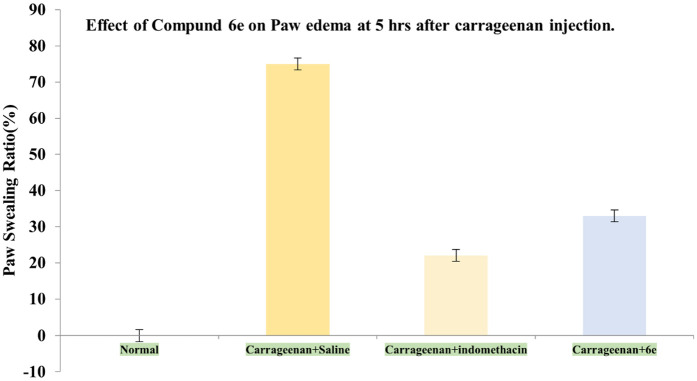
Effect of treated with compound 6e on carrageenan-induced paw edema in rat. The paw swelling ratio is the percent increase of paw volume. Compound **6e** significantly inhibited the ratio of paw swelling at 5h after carrageenan injection compared to positive control Indomethacin. Each value is expressed as mean ± SD (p < 0.0001 versus control, n = 3).

This study investigated the anti-edema effect of compound **6e** using a carrageenan-induced paw edema model in rats. This model is a well-established method for evaluating the anti-inflammatory potential of compounds, as carrageenan injection induces a localized inflammatory response characterized by significant paw swelling. The Fig 29 presents representative photographs of rats from each treatment group, visually demonstrating the effect of compound **6e** on paw edema. The photographs clearly show a comparison between the negative control group (untreated = carrageenan+saline), the positive control group (treated = carrageenan+Indomethacin), and the compound **6e** treatment group. A clear visual reduction in paw swelling in the compound **6e** treated group compared to the control group which support the hypothesis that compound **6e** possesses anti-inflammatory properties. The degree of swelling reduction qualitatively assessed and compared to the positive control. Moreover, H and E staining representative rate changes of each group showing the effect of treatment on paw edema treated with compound **6e** as shown in Fig 30. Too, Fig 31 presents the quantitative data on paw edema, specifically the paw swelling ratio (percent increase in paw volume) at 5 hours post carrageenan+saline injection. This time point is chosen because it typically represents the peak of the inflammatory response in this model. The graph clearly showed the paw swelling ratio for the negative control group, the positive control group, and the compound **6e** treatment groups. A statistically significant reduction in the paw swelling ratio in the compound **6e** treated group compared to the control group which provide strong evidence for the anti-edema activity of compound **6e**. Ideally, the data also compared to the efficacy of the positive control to determine the relative potency of compound **6e**. The observed anti-edema effect of compound **6e** suggests its potential to inhibit the inflammatory cascade triggered by carrageenan. Carrageenan-induced paw edema involves the release of various inflammatory mediators, including prostaglandins, leukotrienes, and cytokines. Compound **6e** may exert its anti-inflammatory effect by targeting one or more of these mediators or their signaling pathways. In conclusion, the results presented in Fig 29a and 29b demonstrate that compound **6e** significantly inhibits carrageenan-induced paw edema in rat. This finding suggests that compound **6e** possesses potent anti-inflammatory. Likewise, the comparison to the positive control provides a benchmark for evaluating the relative efficacy of compound **6e**.

## Compound 6e attenuates neutrophil infiltration and MPO in carrageenan-induced paw inflammation

**Fig 32:**
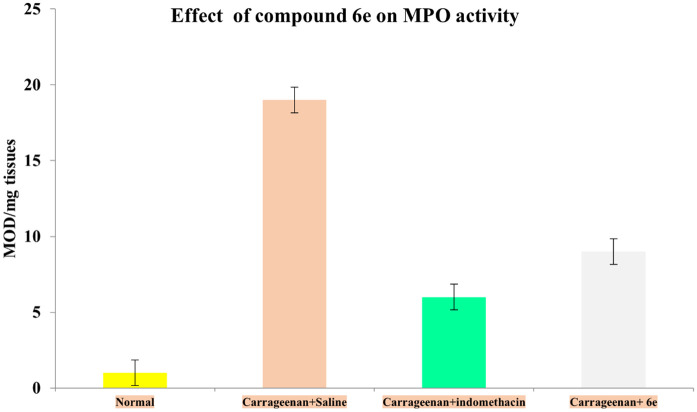
Effect of intraperitoneal administration of compound 6e on myeloperoxidase activity in carrageenan-treated paws. This figure illustrates the impact of compound **6e**, administered intraperitoneally, on myeloperoxidase (MPO) activity in supernatants of homogenates from carrageenan-treated rat paws. MPO activity was assessed 4 hours after carrageenan treatment, highlighting the anti-inflammatory effects of compound **6e** Each value is expressed as mean ± SD (p < 0.0001 versus control, n = 6).

In present study investigated the effect of compound **6e** on neutrophil migration and activity in the carrageenan-induced paw inflammation model in rat. Neutrophil infiltration is a hallmark of the acute inflammatory response, and the activity of the neutrophil-derived enzyme myeloperoxidase (MPO) serves as a reliable indicator of neutrophil accumulation in the inflamed tissue. The Fig 32 demonstrates the impact of compound **6e** on MPO activity in the carrageenan-treated rat paws. The carrageenan injection induced a significant increase in paw MPO activity, reflecting the influx of neutrophils to the site of inflammation. However, treatment with compound **6e** markedly prevented the elevation of MPO activity compared to the carrageenan-treated control group. The reduction in MPO activity observed with compound **6e** administration suggests that the compound **6e** effectively attenuates the recruitment and activation of neutrophils in the inflamed paw tissue. Neutrophils play a crucial role in the early stages of the inflammatory process by releasing a variety of pro-inflammatory mediators and enzymes, such as MPO, implicated in tissue damage and edema formation. Compound 6e treatment appears to reduce the increase in MPO activity, possibly minimizing neutrophil recruitment to the site of inflammation and their activation. This outcome was consistent with previous findings concerning the anti-edema action of compound **6e** in the carrageenan-induced paw edema model, as a reduction in neutrophil-derived inflammatory mediators would likely contribute to the reduction of paw swelling.

The observed inhibition of MPO activity by compound **6e** suggests that the compound **6e** interfere with the signaling pathways and mechanisms that govern neutrophil recruitment and activation in the inflamed tissue. This involves the modulation of chemokines, adhesion molecules, or other mediators that regulate neutrophil migration and degranulation. Furthermore, the reduction in neutrophil-derived MPO activity may have broader implications for the resolution of inflammation. Myeloperoxidase serves not only as an indicator of neutrophil infiltration but also contributes directly to tissue injury and the maintenance of the inflammatory response via the production of oxidative stress and inflammatory lipid mediators. The modulation of MPO activity by compound **6e** may aid in alleviating the adverse consequences associated with neutrophil-mediated inflammation, which could subsequently enhance tissue repair processes and the reestablishment of homeostasis. The beneficial property of compound **6e** is of particular pertinence to the treatment of inflammatory diseases characterized by increased recruitment and activation of neutrophils, such as but not limited to rheumatoid arthritis, acute lung injury, and ischemia-reperfusion injury. In sum, the data shown in Fig 31 demonstrate that compound **6e** considerably reduces myeloperoxidase activity in the carrageenan-induced model of paw inflammation. The observed decrease in MPO activity suggests that compound **6e** might be able to reduce effectively not only the infiltration but also the activation of neutrophils at the inflammatory site. Results like these add further knowledge regarding anti-inflammatory mechanisms associated with compound **6e** and further emphasize its potential therapeutic uses in the treatment of acute and chronic inflammatory diseases.

## Conclusion

This study has succeeded in creating, making, and testing a group of new morpholine-substituted phenoxyacetohydrazide compounds, especially compound **6e**, both contributing to its enhanced pharmacokinetic and anti-inflammatory properties. The morpholine ring offers conformational rigidity and favorable absorption-distribution characteristics, while the fluorophenoxy moiety improves membrane permeability and potential binding affinity through lipophilic interactions and possible π-π stacking within the COX enzyme binding sites. for their ability to reduce inflammation and prevent new blood vessel formation. The experimental results showed significant anti-inflammatory (IC50 = 155 μg/mL) and anti-angiogenic effects (CAM and corneal neovascularization models). This strongly supported our design hypothesis that morpholine substitution enhances biological activity due to increased affinity toward key proteins like VEGF and COX enzymes, as assumed by our molecular docking results. Docking simulations revealed strong binding affinity scores for compound **6e** against VEGF (−13.1622 kcal/mol), COX-1 (−12.5301 kcal/mol), and COX-2 (−12.6705 kcal/mol). Key interactions identified included hydrogen bonding with critical residues such as Arg120 (COX-1), Arg513 (COX-2), and hydrophobic interactions within VEGF binding sites. These molecular-level insights directly correlate with our experimental results, highlighting the compound’s potent anti-inflammatory and anti-angiogenic activity. In carrageenan-induced paw edema, compound **6e** decreased swelling by inhibiting PGE₂-dependent vascular permeability. This confirms its ability to block acute inflammatory responses mediated by COX/prostaglandin pathways. The membrane stabilization test for HRBC, a laboratory measure of anti-inflammation activity, indicated an “IC50” for compound **6e**, which indicate that the compound **6e** protected human red blood cells from hypotonic lysis suggesting stabilization of inflammatory cell membranes—a secondary anti-inflammatory mechanism. Further investigations using in vivo and ex CAM assays showed that compound **6e** effectively suppressed VEGF-induced angiogenesis in a dose-dependent manner. The compound **6e** treatment markedly reduced the microvessel density and total vessel length, corroborating its potent anti-angiogenic properties. In the rat model of alkali-induced corneal neovascularization, compound **6e** treatment significantly reduced the formation of new blood vessels, further confirming its anti-angiogenic effects. Moreover, compound **6e** showed anti-edema effects in the carrageenan-induced paw edema model in rats and was found to attenuate neutrophil infiltration and myeloperoxidase activity in the inflamed paw tissue. These results suggest that compound **6e** possesses anti-inflammatory and anti-angiogenic activities, which may render it a potential candidate for the treatment of diseases characterized by chronic inflammation and pathological angiogenesis. This study points out the promise of carefully designed phenoxyacetohydrazide derivatives as treatments that target multiple pathways. “SAR” analysis indicated that morpholine ring, benzyl group, butanoyl chain, and fluorophenoxy moiety might be important for the favorable pharmacological profile of compound **6e**. This structure may stabilize its interaction with VEGF, COX-1, and COX-2 by forming a favorable hydrogen bonding and electrostatic interaction pattern, thereby enhancing the biological activities of these proteins and hence producing anti-inflammatory and anti-angiogenic effects. In summary, the compound **6e** exhibited promising anti-inflammatory and anti-angiogenic activities potentially through by inhibiting pro-inflammatory prostaglandins (PGE₂), suppressing cytokine-driven NF-κB activation (IL-6 and TNF-α), and modulating angiogenesis-related pathways, as supported by literature and in silico docking studies. The dual anti-inflammatory and anti-angiogenic efficacy of compound **6e** positions it as a promising candidate for ocular and systemic inflammatory disorders. However, further experimental validation by using ELISA and Western blot assays, as well as *in vivo* pharmacokinetic profiling, to confirm the safety and efficacy of compound **6e** as a candidate for anti-inflammatory drug development.

## Supporting information

S1 File Supplemental 1S, 2S and 3S Tables. (DOCX)
