# Correction: Synthesis of novel phenoxyacetohydrazide compounds and evaluation therapeutic potential exhibit as anti-inflammatory and anti-angiogenic

**DOI:** 10.1371/journal.pone.0339331

**Published:** 2025-12-18

**Authors:** Yasser Hussein Issa Mohammed, Ahmed Hassen Shntaif, Saad Alghamdi, Ahd A. Mansour, Naeem F. Qusty, Azhar S. Sindi, Ahmad O. Babalghith, Ghazi A. Bamagous, Eman Adnan Abu-Seer

There is an error in affiliation 3 for author Saad Alghamdi. The correct affiliation 3 is: Department of Clinical Laboratory Sciences, Faculty of Applied Medical Sciences, Umm Al-Qura University, Makkah, Saudi Arabia.
