# Supplementary material for: Synthesis of novel phenoxyacetohydrazide compounds and evaluation therapeutic potential exhibit as anti-inflammatory and anti-angiogenic
Source: PLoS One. 2025 Sep 26;20(9):e0330731. doi: 10.1371/journal.pone.0330731 (PMC12469358; doi:10.1371/journal.pone.0330731)
Supplement: S1 File — (DOCX) [file pone.0330731.s001.docx]

**Synthesis of Novel Phenoxyacetohydrazide Compounds and Evaluation Therapeutic Potential Exhibit as Anti-Inflammatory and Anti-Angiogenic**

Yasser Hussein Issa Mohammed^1¶^*, Ahmed Hassen Shntaif^2¶^, Saad Alghamdi^3¶^, Ahd A. Mansour^4&^, Naeem F Qusty^5&^, Azhar S. Sindi^5&^, Ahmad O. Babalghith^6&^, Ghazi A. Bamagous^7&^ and Eman Adnan Abu-Seer^8&^

**^1^**Department of Pharmacy, Collage of Medicine and Health Science, Hajjah University, Hajjah, Yemen.

**^2^** Department of Chemistry, College of Science for Women, University of Babylon, Alhilla, Iraq.

**^3^**Laboratory Medicine Department, Faculty of Applied Medical Sciences, Umm Al-Qura University, Makkah, Saudi Arabia.

**^4^**Medical Laboratory Science Department, Fakeeh College for Medical Sciences, Jeddah, Saudi Arabia.

**^5^**Department of Clinical Laboratory Sciences, Faculty of Applied Medical Sciences, Umm Al-Qura University, Makkah, Saudi Arabia.

**^6^**Medical genetics Department College of Medicine Umm alqura university, Makkah, Saudi Arabia

**^7^**Department of Pharmacology and Toxicology, Faculty of Medicine, Umm Al-Qura University, Makkah, Saudi Arabia.

**^8^**Epidemiology department, Faculty of Public Health and Health informatics, Umm Al-Qura University, Makkah, Saudi Arabia.

* Corresponding author

E-mail: [issayasser16@gmail.com](mailto:issayasser16@gmail.com)

**Table 1S:**  Docking Score Outcomes for the Interaction Between Compound **6e** and the VEGF Receptor a detailed presentation of the docking results, highlighting the binding affinity and interaction efficiency of compound **6e** with the VEGF receptor.

| **mol** | **S** | **E_conf** | **E_place** | **E_score1** |
| --- | --- | --- | --- | --- |
| **6a** | -12.3228 | 3.6064 | -112.381 | -12.3228 |
| **6a** | -12.0189 | 4 | -96.5441 | -12.0189 |
| **6a** | -11.908 | 1 | -97.196 | -11.908 |
| **6a** | -11.8947 | 3.4 | -82.391 | -11.8947 |
| **6a** | -11.8497 | 3.8 | -123.3102 | -11.8497 |
| **6a** | -11.8487 | 3.8 | -103.5165 | -11.8487 |
| **6a** | -11.8221 | 1.4 | -119.4883 | -11.8221 |
| **6a** | -11.703 | 1 | -99.3086 | -11.703 |
| **6a** | -11.6659 | 2.4001 | -120.8605 | -11.6659 |
| **6a** | -11.6607 | 0.8 | -104.6107 | -11.6607 |
| **6b** | -12.1648 | 2.8134 | -52.4626 | -12.1648 |
| **6b** | -11.8592 | 0.9011 | -83.9419 | -11.8592 |
| **6b** | -11.7422 | 0.8 | -109.2389 | -11.7422 |
| **6b** | -11.7411 | 2.6 | -60.4393 | -11.7411 |
| **6b** | -11.5791 | 0.4 | -104.4965 | -11.5791 |
| **6b** | -11.5686 | 1.3733 | -107.011 | -11.5686 |
| **6b** | -11.5175 | 2.2 | -96.2737 | -11.5175 |
| **6b** | -11.49 | 1.6 | -85.2248 | -11.49 |
| **6b** | -11.4779 | 0.8 | -92.1421 | -11.4779 |
| **6b** | -11.4099 | 0.8 | -113.1303 | -11.4099 |
| **6c** | -12.6999 | 1.4 | -91.6524 | -12.6999 |
| **6c** | -12.2846 | 2 | -92.6482 | -12.2846 |
| **6c** | -12.2785 | 2 | -79.8697 | -12.2785 |
| **6c** | -11.5112 | 0.6 | -94.1085 | -11.5112 |
| **6c** | -11.4498 | 2.4 | -115.6584 | -11.4498 |
| **6c** | -11.4287 | 3 | -86.8855 | -11.4287 |
| **6c** | -11.306 | 2.65 | -104.1017 | -11.306 |
| **6c** | -11.2916 | 1.6 | -77.4877 | -11.2916 |
| **6c** | -11.1562 | 3.7451 | -108.5636 | -11.1562 |
| **6c** | -11.0951 | 2 | -97.9772 | -11.0951 |
| **6d** | -11.9802 | 2 | -97.7888 | -11.9802 |
| **6d** | -11.4852 | 1.6 | -88.8552 | -11.4852 |
| **6d** | -11.2538 | 2 | -95.0623 | -11.2538 |
| **6d** | -11.2358 | 3 | -98.8263 | -11.2358 |
| **6d** | -11.2299 | 0.8 | -114.2488 | -11.2299 |
| **6d** | -11.2003 | 2 | -108.3548 | -11.2003 |
| **6d** | -11.0763 | 3 | -101.4884 | -11.0763 |
| **6d** | -10.9869 | 2.6 | -98.4061 | -10.9869 |
| **6e** | **-12.1333** | 2.8 | -103.3604 | -12.1333 |
| **6e** | -12.1223 | 3.5 | -104.2924 | -12.1223 |
| **6e** | -12.0752 | 3 | -103.4033 | -12.0752 |
| **6e** | -11.9947 | 3.1 | -106.3459 | -11.9947 |
| **6e** | -11.9858 | 2.9075 | -97.3185 | -11.9858 |
| **6e** | -11.8942 | 2.5958 | -104.5194 | -11.8942 |
| **6e** | -11.8801 | 3.5 | -101.6848 | -11.8801 |
| **6e** | -11.8376 | 3 | -101.6818 | -11.8376 |
| **6e** | -11.8003 | 3.1 | -101.6385 | -11.8003 |
| **6f** | -11.4751 | 2.6 | -108.6842 | -11.4751 |
| **6f** | -10.9576 | 2.6 | -110.9735 | -10.9576 |
| **6f** | -10.5283 | 2.4 | -78.3366 | -10.5283 |
| **6f** | -10.5221 | 2.2 | -96.2618 | -10.5221 |
| **6f** | -10.4873 | 1.2 | -94.1381 | -10.4873 |
| **6f** | -10.4741 | 2.4083 | -101.5436 | -10.4741 |
| **6f** | -10.3653 | 2.6 | -98.6072 | -10.3653 |
| **6f** | -10.3579 | 3.2471 | -102.1748 | -10.3579 |
| **6f** | -10.2634 | 1.8 | -86.0727 | -10.2634 |
| **6f** | -10.1818 | 3.225 | -77.6662 | -10.1818 |
| **6g** | -12.5144 | 2.2604 | -125.1495 | -12.5144 |
| **6g** | -12.3905 | 2.6 | -119.1449 | -12.3905 |
| **6g** | -12.1093 | 2.4 | -108.5225 | -12.1093 |
| **6g** | -12.093 | 2.6 | -79.8427 | -12.093 |
| **6g** | -11.8698 | 1.8 | -95.2041 | -11.8698 |
| **6g** | -11.8347 | 2.6 | -106.1633 | -11.8347 |
| **6g** | -11.7584 | 4 | -96.5901 | -11.7584 |
| **6g** | -11.7543 | 3.6 | -100.0301 | -11.7543 |
| **6g** | -11.6983 | 3.6 | -96.2365 | -11.6983 |
| **6g** | -11.6253 | 3.4345 | -97.6814 | -11.6253 |
| **6h** | -11.8104 | 2 | -110.8532 | -11.8104 |
| **6h** | -11.7836 | 2 | -105.3184 | -11.7836 |
| **6h** | -11.7226 | 3 | -103.1174 | -11.7226 |
| **6h** | -11.7203 | 3.8 | -107.0852 | -11.7203 |
| **6h** | -11.5475 | 1.6 | -100.2175 | -11.5475 |
| **6h** | -11.3297 | 2.8 | -85.1081 | -11.3297 |
| **6h** | -11.2804 | 3.8 | -100.8521 | -11.2804 |
| **Indomethacin** | **-9.2719** | 2.4227 | -43.053 | -9.2719 |
| **Indomethacin** | -9.141 | 1 | -40.8237 | -9.141 |
| **Indomethacin** | -9.0714 | 2.6 | -57.5426 | -9.0714 |
| **Indomethacin** | -9.0681 | 2.4208 | -39.205 | -9.0681 |
| **Indomethacin** | -8.9816 | 1.8 | -37.1487 | -8.9816 |
| **Indomethacin** | -8.9448 | 2.6 | -38.2758 | -8.9448 |
| **Indomethacin** | -8.8448 | 2 | -41.6286 | -8.8448 |
| **Indomethacin** | -8.7196 | 2.6007 | -41.6972 | -8.7196 |
| **Indomethacin** | -8.6852 | 1.2 | -39.5414 | -8.6852 |
| **Indomethacin** | -8.6329 | 1.6 | -35.8872 | -8.6329 |

**Table 2S:**  Docking Score Outcomes for the Interaction Between Compound **6e** and the COX-1 Receptor, a comprehensive summary of docking results, highlighting the binding affinity and interaction efficiency of compound **6e** with the COX-1 receptor

| **mol** | **S**  (kcal/mol) | **E_conf** | **E_place** | **E_score1** (kcal/mol) |
| --- | --- | --- | --- | --- |
| **6a** | -11.0778 | 1.0 | -82.9376 | -11.0778 |
| **6a** | -11.0193 | 2.6 | -85.4465 | -11.0193 |
| **6a** | -10.8755 | 3.4 | -113.591 | -10.8755 |
| **6a** | -10.6485 | 3.4 | -78.7383 | -10.6485 |
| **6a** | -10.5867 | 2.8 | -84.996 | -10.5867 |
| **6a** | -10.5846 | 1.2 | -86.0382 | -10.5846 |
| **6a** | -10.4757 | 1.6 | -94.9285 | -10.4757 |
| **6a** | -10.4288 | 0.0 | -93.8905 | -10.4288 |
| **6a** | -10.2088 | 1.0 | -81.6224 | -10.2088 |
| **6a** | -10.1773 | 2.2 | -88.7125 | -10.1773 |
| **6b** | -11.2639 | 1.0 | -70.0857 | -11.2639 |
| **6b** | -10.9586 | 1.4002 | -86.3852 | -10.9586 |
| **6b** | -10.842 | 1.8 | -83.1682 | -10.842 |
| **6b** | -10.5025 | 1.2 | -65.0899 | -10.5025 |
| **6b** | -10.396 | 3.4 | -94.6242 | -10.396 |
| **6b** | -10.1064 | 2.6 | -79.2967 | -10.1064 |
| **6b** | -10.0843 | 3.4 | -94.3856 | -10.0843 |
| **6b** | -10.0532 | 2.8 | -67.8781 | -10.0532 |
| **6b** | -10.025 | 3.0 | -71.5187 | -10.025 |
| **6c** | -9.9101 | 2.8 | -66.1445 | -9.9101 |
| **6c** | -10.1025 | 0.8 | -60.559 | -10.1025 |
| **6c** | -10.0674 | 1.8 | -82.6371 | -10.0674 |
| **6c** | -9.9409 | 2.2 | -89.0847 | -9.9409 |
| **6c** | -9.9112 | 2.2 | -78.0799 | -9.9112 |
| **6c** | -9.8817 | 1.2 | -88.8794 | -9.8817 |
| **6c** | -9.8766 | 3.0 | -75.0786 | -9.8766 |
| **6c** | -9.8535 | 3.6 | -61.5224 | -9.8535 |
| **6c** | -9.8444 | 1.4 | -54.1212 | -9.8444 |
| **6c** | -9.8164 | 1.4 | -53.7747 | -9.8164 |
| **6c** | -9.7435 | 1.8 | -110.3678 | -9.7435 |
| **6d** | -10.8253 | 2.8022 | -61.0985 | -10.8253 |
| **6d** | -10.5698 | 1.8004 | -86.8188 | -10.5698 |
| **6d** | -10.5634 | 1.0 | -85.8663 | -10.5634 |
| **6d** | -10.3925 | 2.0 | -55.0924 | -10.3925 |
| **6d** | -10.2603 | 2.2 | -80.5896 | -10.2603 |
| **6d** | -10.1534 | 1.8004 | -94.1926 | -10.1534 |
| **6d** | -9.9401 | 4.1655 | -83.0011 | -9.9401 |
| **6d** | -9.9355 | 1.4004 | -95.0624 | -9.9355 |
| **6d** | -9.7604 | 2.8 | -75.4774 | -9.7604 |
| **6d** | -9.7055 | 2.6 | -78.2284 | -9.7055 |
| **6e** | **-12.5301** | 2.7555 | -87.6671 | **-12.5301** |
| **6e** | -11.0826 | 1.5505 | -108.1817 | -11.0826 |
| **6e** | -11.0682 | 3.9505 | -92.9193 | -11.0682 |
| **6e** | -11.0535 | 3.7505 | -94.1877 | -11.0535 |
| **6e** | -10.8089 | 1.9505 | -85.5429 | -10.8089 |
| **6e** | -10.7791 | 3.5505 | -92.7377 | -10.7791 |
| **6e** | -10.6623 | 1.8942 | -86.431 | -10.6623 |
| **6e** | -10.509 | 1.3505 | -94.9025 | -10.509 |
| **6e** | -10.4316 | 0.5505 | -58.2903 | -10.4316 |
| **6e** | -10.4214 | 1.9305 | -82.0249 | -10.4214 |
| **6f** | -10.6318 | 3.0092 | -104.1271 | -10.6318 |
| **6f** | -10.5701 | 3.2 | -77.1669 | -10.5701 |
| **6f** | -10.51 | 2.2 | -77.3016 | -10.51 |
| **6f** | -10.3739 | 1.8 | -76.6631 | -10.3739 |
| **6f** | -10.199 | 0.4 | -88.3307 | -10.199 |
| **6f** | -10.1739 | 3.8 | -71.4064 | -10.1739 |
| **6f** | -10.0141 | 2.0 | -93.077 | -10.0141 |
| **6f** | -9.981 | 3.8 | -82.1849 | -9.981 |
| **6f** | -9.8295 | 3.2203 | -71.4975 | -9.8295 |
| **6f** | -9.6648 | 3.8001 | -94.9498 | -9.6648 |
| **6g** | -11.6157 | 1.0 | -56.6833 | -11.6157 |
| **6g** | -11.5952 | 4.0 | -89.9674 | -11.5952 |
| **6g** | -11.0176 | 1.8 | -73.7951 | -11.0176 |
| **6g** | -10.9695 | 1.8 | -86.9726 | -10.9695 |
| **6g** | -10.7841 | 2.4 | -88.2506 | -10.7841 |
| **6g** | -10.6946 | 2.0 | -62.6249 | -10.6946 |
| **6g** | -10.6239 | 2.8004 | -66.8994 | -10.6239 |
| **6g** | -10.5748 | 3.4 | -104.2689 | -10.5748 |
| **6g** | -10.2763 | 0.8 | -99.7872 | -10.2763 |
| **6g** | -10.2548 | 2.0 | -78.095 | -10.2548 |
| **6h** | -11.122 | 1.0 | -69.5176 | -11.122 |
| **6h** | -11.0595 | 3.8 | -111.5165 | -11.0595 |
| **6h** | -10.9406 | 2.4 | -97.2814 | -10.9406 |
| **6h** | -10.8603 | 2.6 | -90.4956 | -10.8603 |
| **6h** | -10.6711 | 1.4648 | -101.7504 | -10.6711 |
| **6h** | -10.4923 | 3.8 | -83.5393 | -10.4923 |
| **6h** | -10.4383 | 3.5221 | -112.7274 | -10.4383 |
| **6h** | -10.241 | 2.4 | -89.6069 | -10.241 |
| **6h** | -10.2088 | 0.0656 | -52.9307 | -10.2088 |
| **6h** | -10.0238 | 3.4 | -89.6539 | -10.0238 |
| **Indomethacin** | **-11.7255** | 2.4 | -103.3923 | **-11.7255** |
| **Indomethacin** | -11.6447 | 1.8 | -60.3887 | -11.6447 |
| **Indomethacin** | -11.6373 | 1.8046 | -75.2515 | -11.6373 |
| **Indomethacin** | -11.345 | 1.2 | -71.7782 | -11.345 |
| **Indomethacin** | -11.0848 | 3.4 | -59.2722 | -11.0848 |
| **Indomethacin** | -11.0748 | 2.0 | -83.6993 | -11.0748 |
| **Indomethacin** | -11.0536 | 2.2208 | -76.7835 | -11.0536 |
| **Indomethacin** | -10.9899 | 2.7156 | -116.4315 | -10.9899 |
| **Indomethacin** | -10.9815 | 1.4334 | -81.4086 | -10.9815 |
| **Indomethacin** | -10.9813 | 2.6049 | -55.4934 | -10.9813 |

**Table 3S:**  Docking Score Outcomes for the Interaction Between Compound **6e** and the COX-2 Receptor, a detailed summary of docking results, showcasing the binding affinity and interaction efficiency of compound **6e** with the COX-2 receptor,

| **mol** | **S** (kcal/mol) | **E_conf** | **E_place** | **E_score1** |
| --- | --- | --- | --- | --- |
| **6a** | -13.4315 | 2.2 | -88.7172 | -13.4315 |
| **6a** | -13.2091 | 3.0 | -93.5699 | -13.2091 |
| **6a** | -12.4888 | 3.638 | -99.1807 | -12.4888 |
| **6a** | -12.1643 | 2.4 | -80.6348 | -12.1643 |
| **6a** | -12.1329 | 1.4 | -111.5769 | -12.1329 |
| **6a** | -11.7969 | 1.8 | -101.9396 | -11.7969 |
| **6a** | -11.6334 | 3.23 | -87.2615 | -11.6334 |
| **6a** | -11.4178 | 1.4 | -84.3467 | -11.4178 |
| **6a** | -11.3815 | 1.8 | -76.4647 | -11.3815 |
| **6a** | -11.2325 | 1.2 | -98.3525 | -11.2325 |
| **6b** | -12.2653 | 2.6 | -81.3393 | -12.2653 |
| **6b** | -12.1081 | 0.8 | -68.5988 | -12.1081 |
| **6b** | -12.0299 | 2.8572 | -113.9961 | -12.0299 |
| **6b** | -11.9636 | 1.0 | -71.2394 | -11.9636 |
| **6b** | -11.9164 | 1.8572 | -102.2611 | -11.9164 |
| **6b** | -11.8369 | 3.0 | -110.5456 | -11.8369 |
| **6b** | -11.624 | 0.8572 | -72.9668 | -11.624 |
| **6b** | -11.5878 | 1.8572 | -79.1504 | -11.5878 |
| **6b** | -11.334 | 1.0 | -72.9111 | -11.334 |
| **6b** | -11.2911 | 3.6134 | -71.0192 | -11.2911 |
| **6c** | -13.1485 | 1.0889 | -106.0743 | -13.1485 |
| **6c** | -12.6781 | 3.5391 | -99.5388 | -12.6781 |
| **6c** | -12.0303 | 1.2 | -68.0692 | -12.0303 |
| **6c** | -11.6411 | 1.4 | -81.0166 | -11.6411 |
| **6c** | -11.4744 | 1.4 | -70.5907 | -11.4744 |
| **6c** | -11.4349 | 1.4 | -79.1463 | -11.4349 |
| **6c** | -11.3477 | 1.2 | -103.2656 | -11.3477 |
| **6c** | -11.2704 | 2.4 | -100.332 | -11.2704 |
| **6c** | -11.2291 | 1.4 | -113.4476 | -11.2291 |
| **6c** | -10.9616 | 3.85 | -87.0647 | -10.9616 |
| **6d** | -12.0797 | 2.2 | -105.5047 | -12.0797 |
| **6d** | -12.0465 | 0.4 | -74.5183 | -12.0465 |
| **6d** | -11.8739 | 2.2 | -94.5598 | -11.8739 |
| **6d** | -11.6146 | 0.8 | -67.8225 | -11.6146 |
| **6d** | -11.5319 | 1.4 | -80.7357 | -11.5319 |
| **6d** | -11.4857 | 1.6 | -101.6316 | -11.4857 |
| **6d** | -11.4525 | 2.0 | -93.5934 | -11.4525 |
| **6d** | -11.4028 | 4.0022 | -91.3685 | -11.4028 |
| **6d** | -11.2208 | 0.8 | -95.9298 | -11.2208 |
| **6d** | -11.1965 | 2.0 | -98.9506 | -11.1965 |
| **6e** | **-12.6785** | 0.9505 | -102.9712 | **-12.6785** |
| **6e** | -11.5767 | 0.9505 | -78.8472 | -11.5767 |
| **6e** | -11.5724 | 1.9505 | -110.2888 | -11.5724 |
| **6e** | -11.5626 | 2.7505 | -102.5293 | -11.5626 |
| **6e** | -11.5591 | 3.9505 | -98.3425 | -11.5591 |
| **6e** | -11.4754 | 1.5505 | -60.1478 | -11.4754 |
| **6e** | -11.3651 | 1.9505 | -76.8632 | -11.3651 |
| **6e** | -11.3316 | 2.3505 | -88.4753 | -11.3316 |
| **6e** | -11.2675 | 3.3505 | -98.1785 | -11.2675 |
| **6e** | -11.0139 | 1.9505 | -84.7603 | -11.0139 |
| **6f** | -12.8326 | 3.4044 | -96.7618 | -12.8326 |
| **6f** | -12.3182 | 3.2 | -98.9704 | -12.3182 |
| **6f** | -11.2701 | 2.2 | -85.3251 | -11.2701 |
| **6f** | -10.7654 | 1.6001 | -68.818 | -10.7654 |
| **6f** | -10.6308 | 2.8 | -64.8648 | -10.6308 |
| **6f** | -10.6086 | 1.2 | -52.9586 | -10.6086 |
| **6f** | -10.5453 | 1.2 | -107.7607 | -10.5453 |
| **6f** | -10.4525 | 0.8002 | -98.7115 | -10.4525 |
| **6f** | -10.1894 | 1.6002 | -116.3435 | -10.1894 |
| **6f** | -10.1602 | 3.4 | -81.1519 | -10.1602 |
| **6g** | -12.6016 | 1.4 | -90.5898 | -12.6016 |
| **6g** | -12.0864 | 1.2 | -90.632 | -12.0864 |
| **6g** | -11.9538 | 3.6 | -90.1196 | -11.9538 |
| **6g** | -11.8178 | 2.0504 | -76.0382 | -11.8178 |
| **6g** | -11.6108 | 2.0464 | -90.9537 | -11.6108 |
| **6g** | -11.505 | 3.0 | -54.2385 | -11.505 |
| **6g** | -11.404 | 1.4793 | -58.4699 | -11.404 |
| **6g** | -11.101 | 1.6 | -69.4849 | -11.101 |
| **6g** | -11.0506 | 2.5735 | -134.9893 | -11.0506 |
| **6g** | -10.9589 | 2.4344 | -109.8722 | -10.9589 |
| **6h** | -12.4483 | 3.6 | -93.3 | -12.4483 |
| **6h** | -12.4158 | 1.0 | -89.3111 | -12.4158 |
| **6h** | -11.9933 | 1.8 | -90.676 | -11.9933 |
| **6h** | -11.8015 | 3.4 | -88.1399 | -11.8015 |
| **6h** | -11.6328 | 2.8 | -92.0793 | -11.6328 |
| **6h** | -11.6276 | 2.6 | -95.3553 | -11.6276 |
| **6h** | -11.4469 | 3.4 | -61.1207 | -11.4469 |
| **6h** | -11.4155 | 2.0 | -89.0658 | -11.4155 |
| **6h** | -11.3898 | 3.0 | -63.5941 | -11.3898 |
| **6h** | -11.1193 | 2.4 | -88.9899 | -11.1193 |
| **Indomethacin** | **-12.5679** | 0.6 | -84.6219 | **-12.5679** |
| **Indomethacin** | -12.5027 | 2.6227 | -107.4465 | -12.5027 |
| **Indomethacin** | -12.4427 | 2.2 | -91.6827 | -12.4427 |
| **Indomethacin** | -12.3382 | 2.0 | -109.4498 | -12.3382 |
| **Indomethacin** | -12.0954 | 2.0 | -73.1709 | -12.0954 |
| **Indomethacin** | -11.8628 | 2.6674 | -82.3908 | -11.8628 |
| **Indomethacin** | -11.835 | 2.3146 | -71.1957 | -11.835 |
| **Indomethacin** | -11.5934 | 1.4253 | -63.8968 | -11.5934 |
| **Indomethacin** | -11.5861 | 1.4334 | -109.6806 | -11.5861 |
| **Indomethacin** | -11.5848 | 3.0253 | -111.6165 | -11.5848 |
